# Discovery of gefitinib-1,2,3-triazole derivatives against lung cancer via inducing apoptosis and inhibiting the colony formation

**DOI:** 10.1038/s41598-024-60000-1

**Published:** 2024-04-22

**Authors:** En Gao, Ya Wang, Gao-lu Fan, Guiqing Xu, Zi-Yuan Wu, Zi-Jun Liu, Jian-Cheng Liu, Long-Fei Mao, Xixi Hou, Shouhu Li

**Affiliations:** 1 School of Chemistry and Chemical Engineering, Henan Normal University, Xinxiang, 453000 China; 2 State Key Laboratory of Quality Research in Chinese Medicine/Macau Institute for Applied Research in Medicine and Health, Macau University of Science and Technology, Macau, China; 3 Department of Pharmacy, Luoyang Third People’ Hospital, Luoyang, 471000 China; 4 College of Basic Medicine and Forensic Medicine, Henan University of Science and Technology, 263 Kaiyuan Road, Luoyang, 471003 China; 5 Department of Pharmacy, The First Affiliated Hospital, College of Clinical Medicine of Henan University of Science and Technology, Luoyang, 471003 China; 6 School of Pharmacy, Xinxiang University, Xinxiang, 453000 Henan China

**Keywords:** Gefitinib, 1,2,3-triazole, Anticancer drug, Cell apoptosis, Cell migration, Biochemistry, Chemical biology, Drug discovery, Molecular biology

## Abstract

A series of 20 novel gefitinib derivatives incorporating the 1,2,3-triazole moiety were designed and synthesized. The synthesized compounds were evaluated for their potential anticancer activity against EGFR wild-type human non-small cell lung cancer cells (NCI-H1299, A549) and human lung adenocarcinoma cells (NCI-H1437) as non-small cell lung cancer. In comparison to gefitinib, Initial biological assessments revealed that several compounds exhibited potent anti-proliferative activity against these cancer cell lines. Notably, compounds **7a** and **7j** demonstrated the most pronounced effects, with an IC_50_ value of 3.94 ± 0.17 µmol L^−1^ (NCI-H1299), 3.16 ± 0.11 µmol L^−1^ (A549), and 1.83 ± 0.13 µmol L^−1^ (NCI-H1437) for **7a**, and an IC_50_ value of 3.84 ± 0.22 µmol L^−1^ (NCI-H1299), 3.86 ± 0.38 µmol L^−1^ (A549), and 1.69 ± 0.25 µmol L^−1^ (NCI-H1437) for **7j**. These two compounds could inhibit the colony formation and migration ability of H1299 cells, and induce apoptosis in H1299 cells. Acute toxicity experiments on mice demonstrated that compound **7a** exhibited low toxicity in mice. Based on these results, it is proposed that **7a** and **7j** could potentially be developed as novel drugs for the treatment of lung cancer.

## Introduction

**Figure 1:**
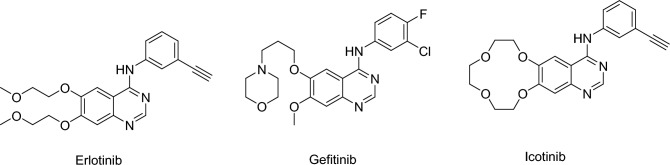
The structures of erlotinib, gefitinib and icotinib.

Cancer remains a critical illness that poses a significant threat to global public health. The treatment and prevention of cancer have consistently been formidable challenges for the medical community. Researchers have conducted comprehensive studies on oncogenes and tumor suppressor genes at the molecular level, leading to the development of novel drugs that target specific molecular markers in cancer cells. This approach has become a key focus in the development of anti-cancer therapeutics^1^. Tyrosine kinases are essential in cellular signal transduction, and there is increasing interest in developing drugs that target this pathway. Lung cancer is the most common malignant tumor in the world, with the epidermal growth factor receptor (EGFR) gene playing a crucial role in the development of lung cancer^2–4^. In recent times, targeted drugs against EGFR (such as gefitinib and erlotinib) have been launched sequentially (Fig. 1), offering fresh hope to individuals suffering from lung cancer^5,6^.

Protein tyrosine kinases are closely related to the occurrence and development of tumors. Overactive tyrosine kinases lead to abnormal activation of downstream signaling pathways, resulting in cell transformation, proliferation, resistance to apoptosis, and promotion of cell growth, ultimately leading to the formation of tumors^7–9^. The clinical trial results indicate that gefitinib belongs to the first generation of targeted therapeutic drugs, which can bind to the epidermal growth factor receptor, and has a good therapeutic effect on non-small cell lung cancer, breast cancer, etc^6^.

EGFR-TKIs exhibit a robust inhibitory effect on EGFR mutant lung cancer cells; however, their inhibitory activity against wild-type lung cancer cells is comparatively weaker. The objective of this study is to explore structural modifications of EGFR-TKIs in order to augment their inhibitory activity specifically against wild-type lung cancer cells.

The 1,2,3-triazole moiety a nitrogenous heterocyclic compound consisting of a five-membered ring. It has found widespread use in various fields such as medicine, pesticides, and more, owing to its ability to serve as a bioisosteric replacement for diverse structures including acyl groups, carboxylic acid groups, and heterocyclic groups. These compounds can bind to tumor cells through various non-covalent interactions such as hydrogen bonding, van der Waals forces, and metal chelation. They induce tumor cell apoptosis through multiple mechanisms, including blocking the growth cycle, inhibiting cell proliferation, and reducing mitochondrial transmembrane potential. These compounds have potential anti-tumor activity.

**Figure 2:**
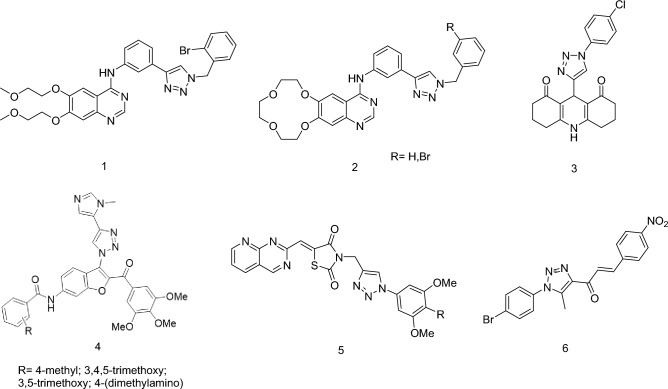
The structures of 1,2,3-triazole derivatives.

Furthermore, utilizing 1,2,3-triazole as a substitute structure has emerged as an effective strategy to overcome patent limitations, fostering imitation innovation. Numerous compounds incorporating the 1,2,3-triazole motif have been developed or investigated for their antitumor, antiviral, and antibacterial biological activities^10–12^. The copper-catalyzed azide-alkyne cycloaddition (Cu-AAC, “click chemistry”) has been employed for the synthesis of 1,2,3-triazole compounds^13^. Researchers have successfully introduced 1,2,3-triazole into the terminal alkyne groups of erlotinib (Fig. 2, 1) or icotinib (Fig. 2, 2), yielding derivatives with significant IDO1 inhibition activity^14,15^. These compounds possess two or more pharmacophores, enabling them to interact with multiple targets in tumor cells simultaneously. This characteristic enhances their anti-tumor activity, reduces side effects, overcomes drug resistance, and improves pharmacokinetics.

**Figure 3:**
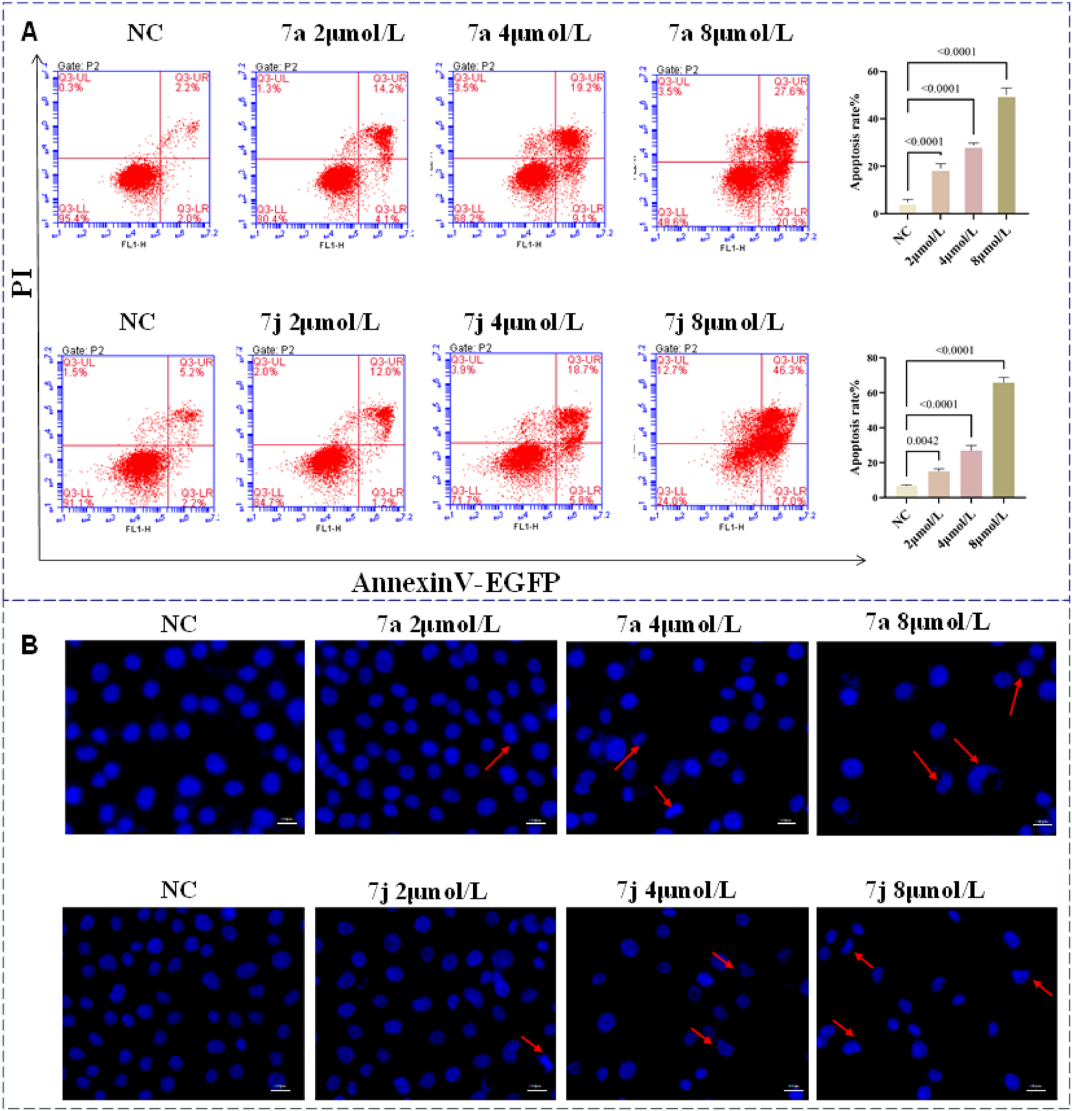
Cell apoptosis induced by **7a** and **7j** in H1299 cells. (**A**) Apoptosis quantification detected by Annexin V-EGFP/PI staining. (**B**) Cell apoptosis morphological changes detected by DAPI staining. Data were mean ± SD. n = 3 for each concentration.

**Figure 4:**
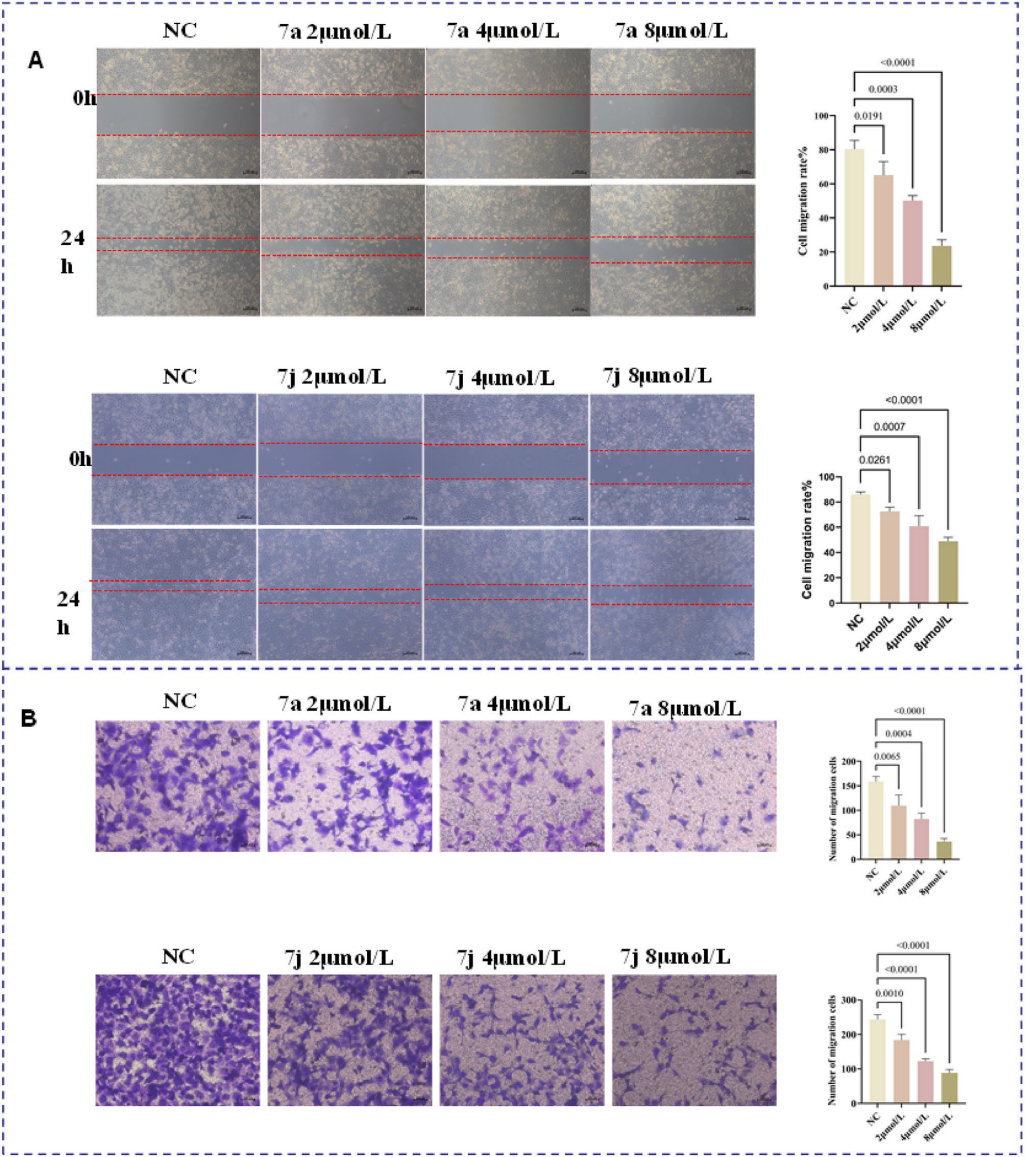
Cell migration and invasion inhibition induced by **7a** and **7j** in H1299 cells. (**A**) Photographs of cells at 0 and 24 h after treatment with different concentrations of drugs (scale bar = 100 μm). (**B**) Cell migration and invasion inhibition induced by **7a** and **7j** in H1299 cells.

**Figure 5:**
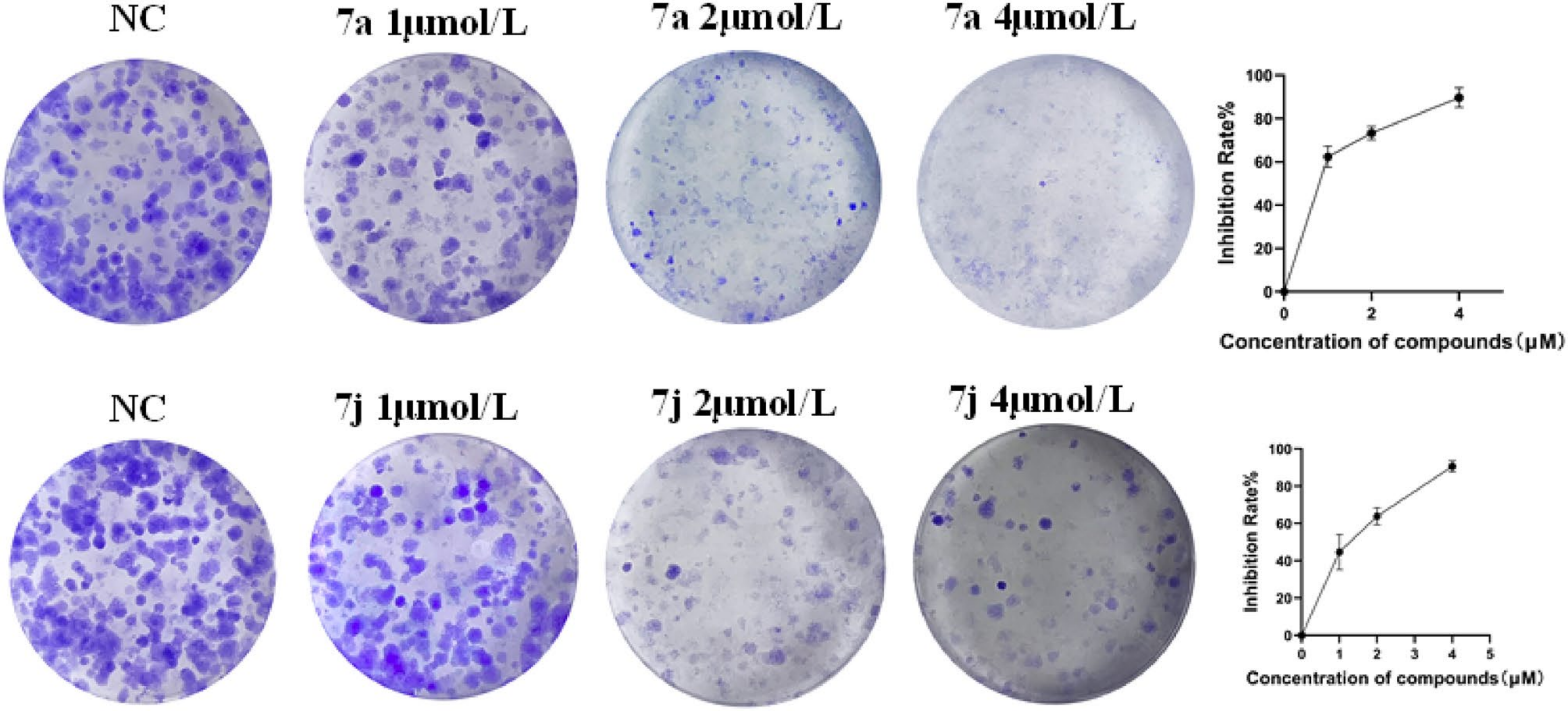
Colony formation pictures of the H1299 treated with **7a** or **7j**.

For instance, the CDK4/6 inhibitor with a 1,2,3-triazole-quinoline-2,4-dione structure (Fig. 2, 3) has been found to exhibit significant inhibitory activity against breast cancer cell lines, including MCF-7, MDA-MB-231, BT-474, and SK-BR3, comparable to that of abemaciclib. This compound arrests the G1/S cell cycle transition by blocking Rb phosphorylation. Pharmacokinetic studies demonstrate a longer plasma half-life and lower plasma clearance compared to abemaciclib, effectively controlling tumor growth volume while minimizing side effects^16^. In addition, further research has been conducted on the synthesis of acylamide derivatives of 1,2,3-triazole-benzofuran compounds (Fig. 2, 4). These derivatives have demonstrated potential anti-cancer activity against various types of cancer cell lines such as prostate cancer (PC3), lung cancer (A549), breast cancer (MCF7), and ovarian cancer (A2780). Molecular docking studies have supported the predicted binding affinity, indicating enhanced inhibitory effects^17^. Similarly, a novel pyrido[2,3-d]pyrimidine-thiazole-1,2,3-triazole compound (Fig. 2, 5) has been designed and synthesized, showing inhibitory effects on EGFR kinase and anti-proliferative activity in different cell lines^18^.

**Figure 6:**
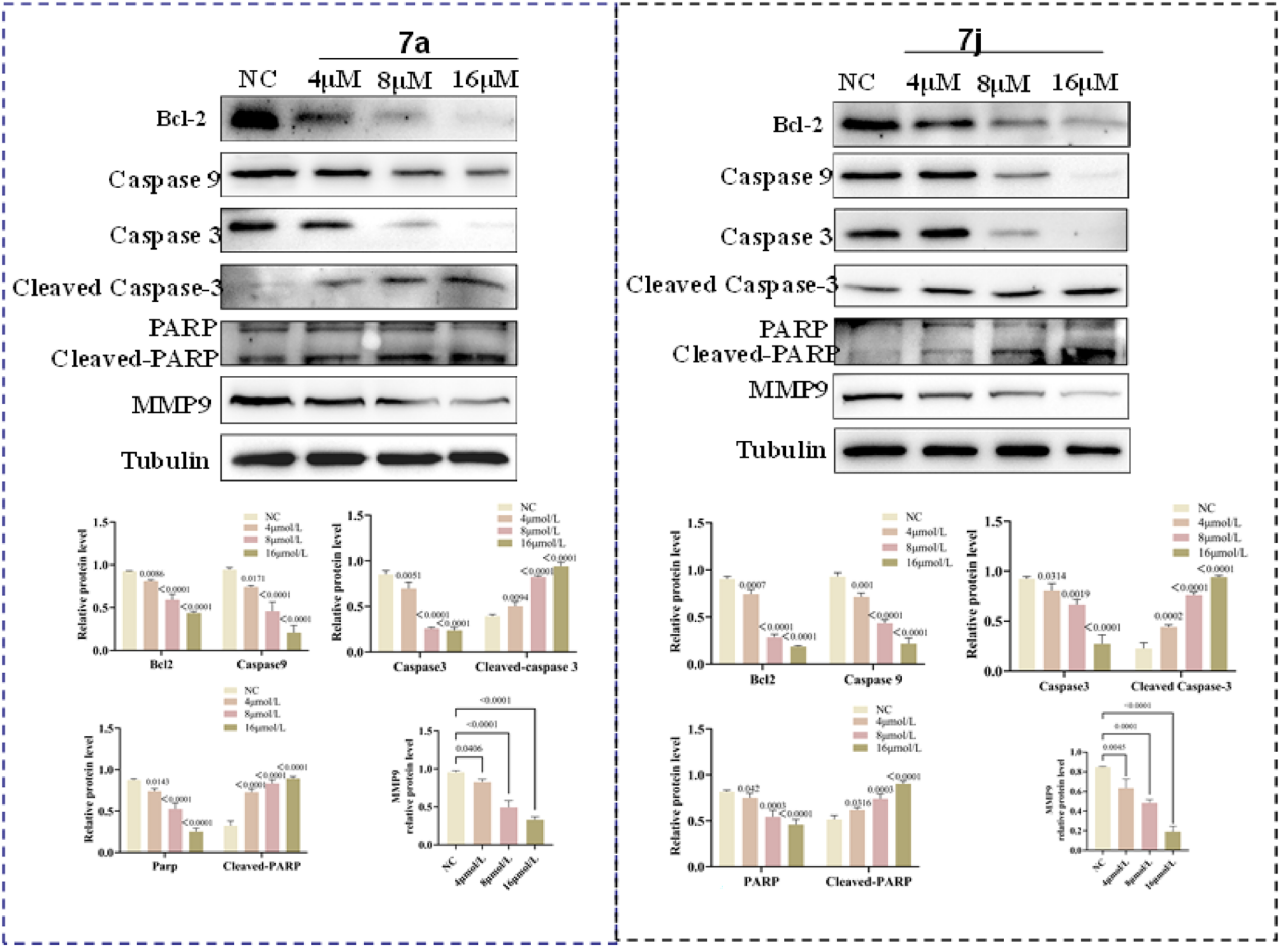
Assessment of apoptosis and migration induced by **7a** and **7j**. (**A**) Western blotting results of the protein levels in H1299 cells treated with 4, 8 or 16 μM. compounds **7a**. Numbers below each lane indicated the relative expression level of the protein. (**B**) Western blotting results of the protein levels in H1299 cells treated with 4, 8 or 16 μM. compounds **7j**. Numbers below each lane indicated the relative expression level of the protein.

In summary, the integration of 1,2,3-triazole with other pharmacophores represents a highly effective approach in the development of novel anti-cancer drugs (Fig. 2, 6)^19^. The diverse studies presented demonstrate the remarkable potential of 1,2,3-triazole-modified compounds in cancer treatment, offering insights into their mechanisms and promising outcomes in cell-based studies.

To identify compounds with improved inhibitory effects on wild-type lung cancer cells, we modified gefitinib using a click reaction, introducing a 1,2,3-triazole moiety into its structure. We hoped that this modification would exhibit inhibitory effects on wild-type lung cancer cells. Using gefitinib as a positive control, we employed the CCK-8 assay to investigate the in vitro anti-tumor activity on wild-type human non-small cell lung cancer cells (NCI-H1299, A549) and human lung adenocarcinoma cells (NCI-1437). Furthermore, we studied the impact on tumor cell colony formation, migration ability, and apoptosis.

## Chemistry

**Scheme 1:**
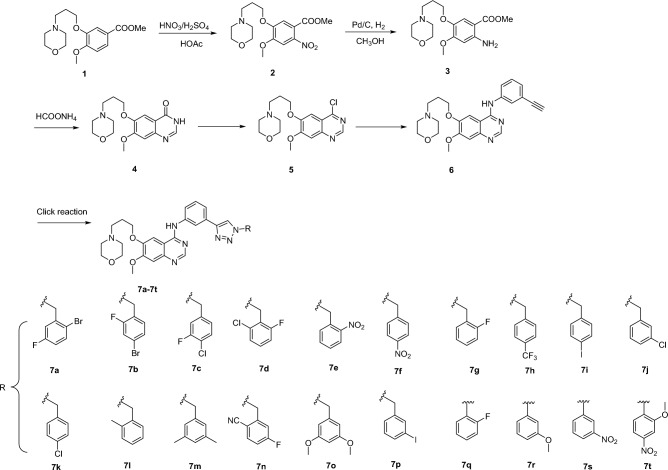
Synthesis route of Gefitinib-1,2,3-triazole derivatives (compounds **7**).

The synthetic strategy for the preparation of the target molecules is illustrated through a literature review in Scheme 1^20–22^. Nitration of **1** was realized using HNO_3_/H_2_SO_4_ as the nitration agent, and the resulted compound **2** was readily converted to compound **3** under the conventional hydrogenation conditions. Cyclization reaction between **3** and formamide produced compound **4**. The chlorination of hydroxyl group with phosphorus oxychloride produced compound **5**. Compound **6** was obtained after reaction of **5** and 3-aminophenylacetylene. Finally, 1,2,3-triazole gefitinib derivatives **7a-7t** was synthesized through a copper(I)-catalyzed azide-alkyne cycloaddition reaction. The reaction conditions of these operations were mild and easy to control. The structures of the key intermediates and all target compounds were confirmed through nuclear magnetic resonance spectroscopy (^1^H NMR and ^13^C NMR) and high-resolution mass spectrometry (HRMS). The detail and figures of NMR spectrums and HRMS were included in the supplementary files.

## Results and discussion

### Compounds 7a-7t suppressed cancer cells viability

In this study, the CCK-8 assay was employed to investigate the cell inhibitory effects of compounds **7a-7t** on human lung cancer cells NCI-H1299, NCI-H1437, and A549, with gefitinib used as a positive control. The results (Table 1) indicated that almost all compounds exhibited inhibitory effects on NCI-H1299, NCI-H1437, and A549 lung cancer cells, with compounds **7a** and **7j** showing the best performance. Specifically, in the NCI-H1299 cell line, the IC_50_ values for **7a** and **7j** were 3.94 ± 0.17 μM and 3.84 ± 0.22 μM, respectively. In the NCI-H1437 cell line, the IC_50_ values for **7a** and **7j** were 1.83 ± 0.13 μM and 1.69 ± 0.25 μM, respectively. In the A549 cell line, the IC_50_ values for **7a** and **7j** were 3.16 ± 0.11 μM and 3.86 ± 0.38 μM, respectively. These values were all superior to the inhibitory effects of gefitinib in the three cell lines (IC_50_ = 14.62 ± 0.90 μM, IC_50_ = 20.56 ± 2.45 μM, and IC_50_ = 14.62 ± 0.43 μM, respectively). The experimental results suggest that the gefitinib derivatives have higher cytotoxicity and play a pro-apoptotic role in lung cancer cells.

**Table 1:** The half-maximal inhibitory concentration (IC_50_) of compounds **7a-7t.**

Compd No.	IC_50_ (μM)		
	H1299	A549	1437
**7a**	3.94 ± 0.17	3.16 ± 0.11	1.83 ± 0.13
**7b**	4.60 ± 0.16	5.28 ± 0.20	3.65 ± 0.04
**7c**	6.24 ± 0.32	5.68 ± 0.14	3.08 ± 0.47
**7d**	6.04 ± 0.41	8.50 ± 0.41	4.83 ± 0.16
**7e**	5.81 ± 0.12	4.38 ± 0.07	2.22 ± 0.18
**7f**	5.65 ± 0.16	16.44 ± 1.07	3.95 ± 0.45
**7g**	5.97 ± 0.36	6.23 ± 0.12	1.66 ± 0.18
**7h**	4.89 ± 0.16	7.13 ± 1.21	5.21 ± 0.30
**7i**	5.60 ± 0.09	5.76 ± 0.62	4.69 ± 0.23
**7j**	3.84 ± 0.22	3.86 ± 0.38	1.69 ± 0.25
**7k**	6.90 ± 0.23	4.91 ± 0.52	2.90 ± 0.23
**7l**	4.92 ± 0.49	6.59 ± 0.20	0.84 ± 0.10
**7m**	4.48 ± 0.41	6.54 ± 0.21	3.72 ± 0.17
**7n**	6.02 ± 0.24	3.78 ± 0.03	4.67 ± 0.28
**7o**	6.78 ± 0.41	12.03 ± 0.15	5.21 ± 0.13
**7p**	5.04 ± 0.33	8.5 ± 0.34	4.93 ± 0.14
**7q**	7.68 ± 0.11	9.03 ± 0.15	7.21 ± 0.10
**7r**	9.35 ± 0.31	12.94 ± 0.25	6.21 ± 0.23
**7s**	9.68 ± 0.21	11.03 ± 0.25	8.21 ± 0.13
**7t**	13.04 ± 0.23	13.50 ± 0.33	14.93 ± 0.14
**Gefitinib**	14.62 ± 0.90	14.62 ± 0.43	20.56 ± 2.45

Compounds **7a** and **7j** exhibited IC_50_ values of 18.87 + 1.03 μM and 17.68 ± 0.52 μM respectively in the L02 cell line. Moreover, our study demonstrated that these compounds are less toxic to normal hepatocytes and more effective in killing lung cancer cells, with a survival rate of normal hepatocytes L02 treated with **7a** and **7j** at a concentration of 4 μM between 70% and 90%. These findings suggest that compounds **7a** and **7j** hold great potential as clinical therapeutic agents for lung cancer (Table 2). The introduction of phenyl or benzyl groups had a notable impact on the activity of the compounds. Notably, when the triazole linkage was benzyl, the activity was superior to phenyl. The study clearly demonstrates the successful structural modification of gefitinib, resulting in significant differences in compound activity. Furthermore, the addition of bromine atoms in the neighbouring position of the benzyl ring or the addition of fluorine or chlorine atoms in the interstitial position significantly improved the in vitro antitumour activity of the compounds.

**Table 2:** Survival rate of **7a** and **7j** in L02 cells.

Compd No.	IC_50_ (μM)	Survival rate %, 48 h				
		2 μM	4 μM	8 μM	16 μM	32 μM
**7a**	18.87 ± 1.03	95.37 ± 2.98	71.97 ± 2.33	66.89 ± 1.52	63.25 ± 1.25	32.43 ± 0.95
**7j**	17.68 ± 0.52	94.49 ± 4.11	89.12 ± 5.50	75.59 ± 2.65	59.14 ± 1.54	24.48 ± 3.68

### Compounds 7a and 7j induce apoptosis in H1299 cells

To elucidate whether the inhibitory effects on cell proliferation and cytotoxicity of these compounds are associated with apoptosis, we conducted relevant experiments focusing on compounds **7a** and **7j**, which demonstrated good inhibitory effects on the proliferation of the three types of cancer cells. H1299 cells were treated with different concentrations of **7a** and **7j** for 48 h. The cells were stained with Annexin-V and PI, and the proportion of apoptotic cells was detected using flow cytometry.

As shown in Fig. 3A, after treatment with compound **7a**, the total apoptotic cell proportions in H1299 were 19.03 ± 2.10% (2 μmol/L), 28.73 ± 1.12% (4 μmol/L), and 50.1 ± 2.91% (8 μmol/L) (Fig. 3). Following treatment with compound **7j**, the total apoptotic cell proportions in H1299 were 14.97 ± 1.54% (2 μmol/L), 27.07 ± 2.77% (4 μmol/L), and 65.77 ± 2.93% (8 μmol/L) (Fig. 3A). Compared to the control group, as the drug concentration increased, the apoptotic proportion gradually increased (*P* < 0.01, *P* < 0.05). These results suggest that both **7a** and **7j** significantly promote apoptosis in the H1299 lung cancer cell line and exhibit concentration-dependent effects.

In order to further verify the apoptotic activity induced by **7a** and **7j** in lung cancer cells, H1299 cells were treated with different concentrations of compounds **7a** and **7j** for 48 h, followed by DAPI staining. The cells were observed under a fluorescence microscope, and representative images are shown in Fig. 3B. Compared to the normal control group, H1299 cells treated with compounds **7a** and **7j** exhibited typical apoptotic features. As the drug concentration increased, nuclear staining intensified, and even nuclear condensation and fragmentation phenomena were observed. Therefore, the apoptosis-related results indicate that both **7a** and **7j** can significantly promote apoptosis in human lung cancer cells (H1299) in a concentration-dependent manner.

### Compounds 7a and 7j suppress metastasis in H1299 cell

To investigate the impact of compounds on the migration of wild-type lung cancer cells, we utilized a wound healing assay and Transwell assay to assess the changes in migration ability of H1299 cells after treatment with compounds **7a** and **7j**. Figure 4 presents the results of the wound healing assay, indicating that after treatment with compound **7a**, the migration closure rates of H1299 cells were 65.16 ± 7.88% (2 μmol/L), 50.12 ± 2.99% (4 μmol/L), and 23.54 ± 3.65% (8 μmol/L). After treatment with compound **7j**, the migration closure rates of H1299 cells were 72.57 ± 3.43% (2 μmol/L), 60.91 ± 8.48% (4 μmol/L), and 48.88 ± 3.23% (8 μmol/L) (Fig. 4A). The migration closure rates in the compound-treated groups were significantly lower than the control group, showing a concentration-dependent trend (*p* < 0.01, *p* < 0.05).

Transwell chamber migration assay is another important method to assess cell migration ability. Tumor cells are seeded in the chamber, and the number of tumor cells that pass through the membrane is counted to evaluate the migration ability of tumor cells^23^. The results of the Transwell migration assay (shown in Fig. 4B) also indicate that compared to the normal control group, the number of cells passing through the membrane to the lower surface of the chamber in the compound-treated group significantly decreased (*p* < 0.01). Therefore, the experiments demonstrate that compounds **7a** and **7j** can inhibit the migration ability of non-small cell lung cancer cells in a concentration-dependent manner.

### Compounds 7a and 7j inhibit the clonogenic ability in H1299 cells

The colony formation assay is an important method for evaluating the ability of adherent cells to proliferate and form colonies on a plate, and it is widely used to assess cell proliferation^24^. We also employed the colony formation assay to further confirm that compounds **7a** and **7j** can inhibit the proliferation of lung cancer cells, as shown in Fig. 5. After treating lung cancer cells with low, medium, and high concentrations (1, 2, 4 μmol/L) of compounds for 7 days, the number of cell colonies was counted to analyze the proliferation inhibition rate. The results showed a significant decrease in the number of cell colonies in the compound-treated groups, and this effect was concentration-dependent. Even at the lowest concentration of 1 μmol/L, the compounds began to markedly inhibit the formation of H1299 cell colonies. Particularly at a concentration of 4 μmol/L, colony formation was almost completely suppressed, indicating that compounds **7a** and **7j** exert a strong anti-proliferative effect on H1299 cells (Fig. 5).

### Compounds 7a and 7j trigger apoptosis via the apoptosis signaling pathway

We conducted an in-depth investigation into the mechanism of apoptosis induced by compounds **7a** and **7j**. The Bcl-2 protein family is located upstream of the apoptosis signaling pathway. Bcl-2 proteins play an anti-apoptotic role by inhibiting the release of cytochrome C (cyt-c) from mitochondria. Previous literature reports that many anticancer drugs induce apoptosis by downregulating Bcl-2 expression^25^. During apoptosis, the expression of Bcl-2 is inhibited, cyt-c is released, and it binds with the apoptotic protease activation factor to form the apoptosome. This triggers a cascade reaction of Caspase family proteins, with apoptosome promoting the activation of Caspase9. Activated Caspase9 then activates Caspase3, forming the Caspase3 cleavage body, which further activates downstream PARP protein, ultimately promoting cell apoptosis^26^. Therefore, by detecting the expression levels of Bcl-2, caspase9, caspase3, and PARP proteins, we found that the treatment with compounds **7a** and **7j** resulted in a decrease in Bcl-2 and caspase9 protein expression levels, while the expression levels of Cleaved-Caspase3 and Cleaved-PARP proteins increased. These results suggest that compounds **7a** and **7j** may effectively induce apoptosis in non-small cell lung cancer cells through the Bcl-2/caspase3/PARP signaling pathway. After treating H1299 cells with different concentrations of compounds for 48 h, cell proteins were extracted, and western blot was used to detect the expression changes of apoptosis-related proteins. The results showed that the migration-related protein MMP9 did not show significant changes in the low drug concentration group but decreased significantly in the high drug concentration group. The apoptosis-related protein Bcl-2 exhibited a decrease in expression with increasing drug concentration, while Cleaved-PARP showed an increase in expression with increasing drug concentration. The expression of caspase3, PARP, and caspase9 proteins decreased with increasing drug concentration, indicating that apoptosis in the high drug concentration group was significantly stronger than that in the low drug concentration group and the control group (*p* < 0.01, *p* < 0.05).

Previous studies have reported that MMP9 plays a crucial role in angiogenesis and cell migration^27^. In non-small cell lung cancer tissues, the protein expression level of MMP9 is significantly higher than in normal adjacent tissues, suggesting a close association between the high expression of MMP9 and the malignant metastasis of lung cancer as well as poor prognosis^28^. To further investigate whether compounds regulate the migration ability of non-small cell lung cancer by modulating MMP9 protein levels, Western Blot results show a decrease in MMP9 protein expression with increasing drug concentration. Therefore, compounds **7a** and **7j** may inhibit the migration ability of non-small cell lung cancer by downregulating MMP9 protein expression (Fig. 6).

### Acute oral toxicity assessment

**Figure 7:**
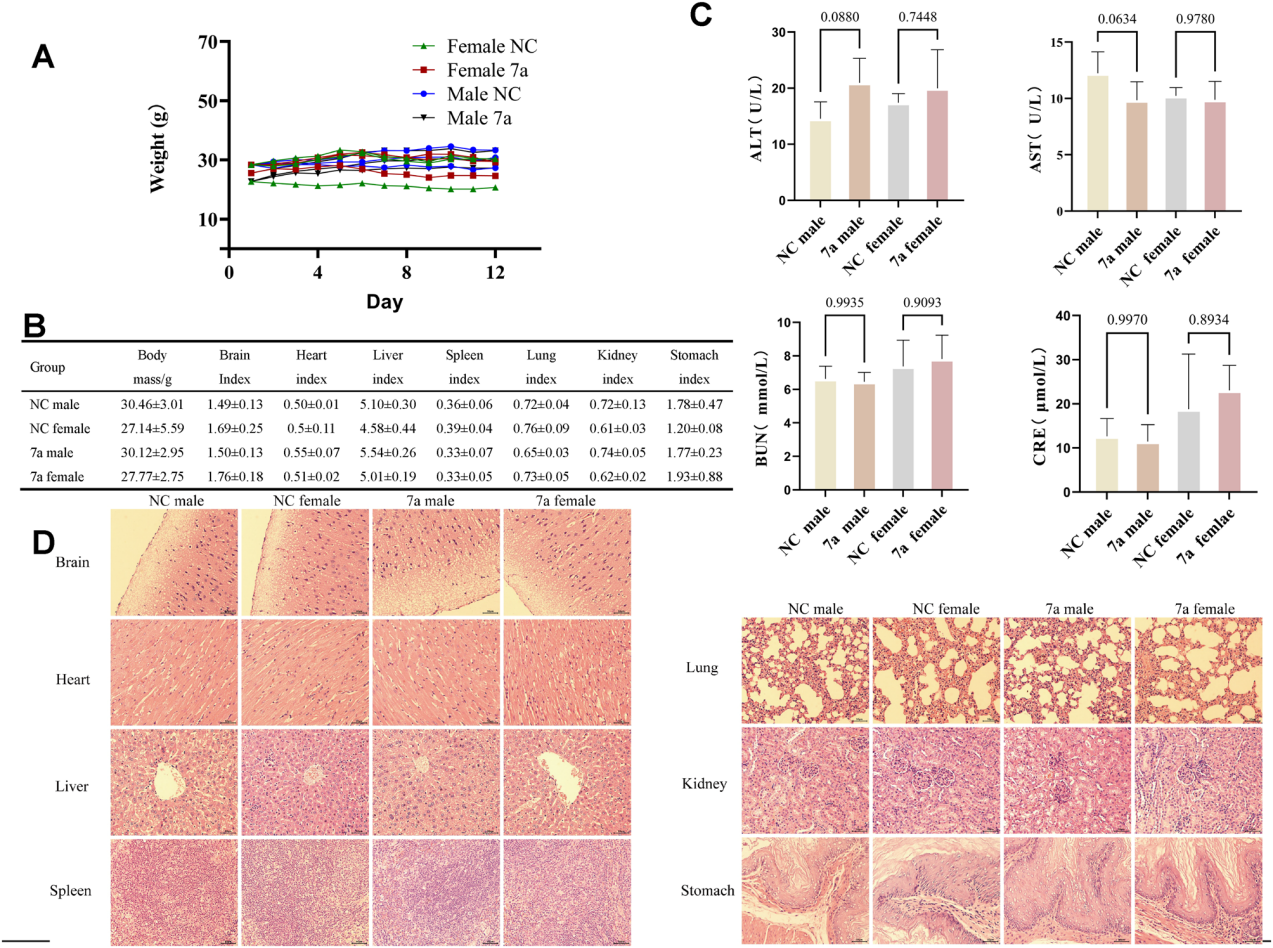
Compound **7a** in vivo toxicity of mice. (**A**) Acute toxicity experiments of compound **7a** were conducted in mice. Mouse body weight changed. (**B**) The acute toxicity experiments examined the effects of compound **7a** on mouse organs (**C**) Effect of acute toxicity experimental studies on blood biochemical indices in mice. (**D**) H&E staining were performed on various organs of mice treated with compound **7a**.

We further evaluated the safety of the compounds through acute toxicity experiments in mice. From the initial gavage to the 12th day, there was no statistical difference in mouse body weight compared to the control group. Observation of organ morphology during dissection revealed no pathological changes, and organ indices showed no statistical differences compared to the control group. Biochemical indicators, including serum alanine aminotransferase (ALT), aspartate aminotransferase (AST), blood urea nitrogen (BUN), and creatinine (CRE), were measured. ALT and AST are commonly used to assess liver function, reflecting physiological and pathological changes in liver function. Elevated levels of ALT and AST in the serum indicate damage to liver cells or mitochondria^29^. BUN and CRE are typically used to reflect renal function, and an increase in their levels in the serum suggests a decline in kidney function or kidney injury^23^. The results of these biochemical indicators showed no statistical differences compared to the control group and were within the normal range. HE staining of the mouse brain, heart, liver, spleen, lungs, kidneys, and stomach further confirmed that mice did not exhibit obvious toxic characteristics at the drug concentration tested. This additional evidence indicates a high level of safety for the compounds, providing experimental data reference for the clinical safety of the compounds (*p* > 0.05) as shown in Fig. 7.

From the day of self-administration until the 12th day, mice in all groups showed no apparent toxic reactions, and there were no deaths. When compared to the control group, there were no significant differences in body weight among the experimental groups. (*p* > 0.05) as shown in Fig. 7.

## Conclusion

In summary, gefitinib derivatives containing a 1,2,3-triazole ring were designed, synthesized, and evaluated for their anti-tumor activity against wild-type lung cancer cells. The synthetic method was simple and efficient, and the compounds were structurally characterized and confirmed. Several of these compounds exhibited superior antitumor activity compared to gefitinib against one or more cancer cell lines employed in this study. Among them, compounds **7a** and **7j** exhibited strong anti-proliferative activity in three non-small cell lung cancer. Through cell function and mechanism studies, it was found that compounds **7a** and **7j** downregulated the expression of Bcl-2, Caspase9, and MMP9 proteins, upregulated the expression of Cleaved-Caspase3 and Cleaved-PARP proteins, inhibited tumor cell proliferation and migration, promoted cell apoptosis. Acute toxicity experiments in mice also confirmed their safety, providing foundational data for future in vivo experiments and clinical applications of these novel gefitinib derivatives, with the hope that these compounds may become effective and low-toxicity anti-cancer drugs.

## Experimental work

### Materials and Chemistry

The gefitinib-1,2,3-triazole derivative was synthesized in-house. All the reagents and solvents used were obtained from a commercially available source. The ^1^H and ^13^C NMR spectra were acquired in a DMSO-*d*_*6*_ solution using a Bruker 400 MHz NMR spectrometer. High-resolution mass spectra (HRMS) measurements were carried out using a Bruker Compact mass spectrometer.

#### Preparation of methyl 4-methoxy-5-(3-morpholinopropoxy)-2-nitrobenzoate (2)

To a mixture of compound** 1** (15 g, 50 mmol) in acetic acid (100 mL) solution was added the mixture of nitric acid (3.92 g, 62 mmol) and sulfuric acid (6.07 g, 62 mmol) dropwise at 5 °C. After addition, the system gradually rose to room temperature, and the mixture was stirred at room temperature for 24 h. the reaction was poured into ice water (100 mL), The precipitate was collected by filtration, washed with water (20 mL) and recrystallized with isopropyl alcohol and dried successively to afford **2** (14 g, 80%). Mp:118–121 °C. ^1^H NMR (400 MHz, CDCl_3_): *δ* 7.45 (s, 1H),7.10 (s, 1H),4.18 (t, 2H), 3.94 (s, 3H), 3.93 (s, 3H), 3.73 (t, 4H), 2.55–2.47 (m, 6H), 2.06 (t, 2H).

#### Preparation of methyl 2-amino-4-methoxy-5-(3-morpholinopropoxy)benzoate (3)

A mixture of compound** 2** (10 g, 28 mmol) and Pd/C (10%wt., 1 g) in methanol (150 mL) was stirred at 25 °C under H_2_ (15 psi) atmosphere for 24 h. The reaction mixture was filtered through celite and the filter cake washed with methanol (150 mL). The filtrate was concentrated to afford **3** (7.70 g, 85%) as a light yellow solid. Mp:89–90 °C. ^1^H NMR (400 MHz, DMSO-*d*_6_): *δ* 7.54(s,1H),7.14(s,1H), 6.43(s,2H), 4.28 (t, 2H), 3.97 (s, 3H), 3.91(s, 3H), 3.63(t, 4H), 2.57–2.52(m, 6H), 2.16–2.15 (m, 2H).

#### Preparation of 7-methoxy-6-(3-morpholinopropoxy)quinazolin-4(3H)-one (4)

A mixture of compound** 3** (4.0 g, 10 mmol) and ammonium formate (2.0 g, 32 mmol) in DMF (30 mL) was stirred at 165 °C in nitrogen environment for 3–4 h. The reaction mixture was poured into ice-water (100 mL) and extracted with ethyl acetate. The combined extracts were washed with sat NaCl (50 mL) and then dried with anhydrous magnesium sulfate, concentrated to afford **4** (3.0 g, 75%) as a yellow solid. Mp:247–250 °C.^1^H NMR (400 MHz, DMSO-*d*_6_) *δ* 7.97 (s, 1H), 7.44 (s, 1H), 7.12 (s, 1H), 4.11 (t, *J* = 6.5 Hz, 2H), 3.90 (s, 3H), 3.58 (t, *J* = 4.6 Hz, 4H), 2.44 (t, *J* = 7.0 Hz, 2H), 2.37 (s, 4H), 1.99–1.80 (m, 2H).

#### 4-(3-((4-chloro-7-methoxyquinazolin-6-yl)oxy)propyl)morpholine (5)

DMF (1.3 g, 18 mmol) was added dropwise over 20 min to a stirred solution of oxalyl chloride (2.28 g, 18 mmol) in DCM (12 mL) under N_2_ at 25 °C, resulting in an exotherm and gas evolution. When gas evolution ceased, compound **4** (2.6 g, 7.8 mmol) was added with mechanical agitation, the mixture was heated to reflux for 24 h and cooled to 25 °C, then the reaction was quenched with dilute aqueous Na_2_HPO_4_ solution (0.5 M, 25 mL). The resulting mixture was stirred on an ice bath for 2 h, and the solid was collected, rinsed with water (25 mL), and dried at 50 °C under vacuum to **5** (2.2 g, 85%) which was used directly. Mp:116–119 °C. ^1^H NMR (400 MHz, DMSO-*d*_6_) *δ* 8.86 (s,1H), 7.43 (s, 1H), 7.38 (s,1H), 4.23 (t, 2H), 3.95 (s, 3H), 3.57 (t, 4H), 2.43 (t, 2H), 2.37 (s, 4H), 2.03–1.92 (m, 2H).

#### Preparation of N-(3-ethynylphenyl)-7-methoxy-6-(3-morpholinopropoxy)quinazolin-4-amine (6)

Reaction of compound **5** (1.5 g, 4.5 mmol) and m-acetylenyl aniline (1.56 g, 13.4 mmol) was refluxed for 6 h in stirred 2-propanol (40 mL) under N_2_ for gave **6** (1.6 g, 65%). ^1^HNMR (400 MHz, DMSO-*d*_6_) *δ* 11.22 (s, 1H), 8.78 (s, 1H), 8.50 (s, 1H), 7.99 (s, 1H), 7.91 (d, J = 8.2 Hz, 1H), 7.48 (t, J = 7.9 Hz, 1H), 7.37 (d, J = 8.8 Hz, 2H), 4.40 (s, 2H), 4.28 (s, 1H), 3.96–3.79 (m, 7H), 3.39–3.160 (m, 6H), 2.35(s, 2H).

#### General procedure for the preparation of compound 7

Aryl-azido (150 mg, 0.7 mmol) and 6 (250 mg, 0.56 mmol) were added to 15 mL mixed solvent (water/tert-butanol/THF = 1:1:1). Copper sulfate pentahydrate (14 mg, 0.1 mmol) and sodium ascorbate (23 mg, 0.1 mmol) were added and the mixture was stirred at 85 °C for 12 h. After the completion of the reaction (monitored by TLC), the mixture was extracted with dichloromethane (15 mL × 3). The combined organic phase was washed successively with brine, dried over sodium sulfate and concentrated in vacuo. The residue was purified by through column chromatography (CH_2_Cl_2_/MeOH = 20:1) to give the desired compound 7a-7t as a crystalline powder.

### Biological study

Lung cell lines A549, NCI-H1437, and NCI-H1299 were purchased from Procell Life Science &Technology Co., Ltd (Wuhan, China). Ham’s F-12 K Medium and Fetal bovine serum (FBS) were purchased from Procell Life Science &Technology Co., Ltd (Wuhan, China). RPMI 1640 Medium and diamidino-phenyl-indole (DAPI) were purchased from Solarbio Science Technology (Beijing, China). Cell Counting Kit-8 and Annexin V-EGFP Apoptosis Detection Kit were obtained from Beyotime Biotechnology (Shanghai, China). The primary antibodies against Caspase3 (1:500), PARP/cleaved-PARP (1:750), and MMP9 (1:1000) were obtained from Wan lei Biotechnology (Shenyang, China). Alpha Tubulin (1:2000), cleaved-Caspase3 (1:500), Caspase9(1:300) was purchased from Proteintech (Wuhan, China). Bcl-2(1:1000), HRP-conjugated affinipure goat anti-rabbit IgG (1:1000) was purchased from Cell Signaling Technology (MA, United States).

#### Cell culture

NCI-H1299 and NCI-H1437 cells were cultured in a humidified incubator at 37 °C with 5% CO_2_ in RPMI-1640 (No.31800, Solarbio) supplemented with 10% fetal bovine serum (FBS), and 1% Penicillin Streptomycin. A549 cells were cultured in a humidified incubator at 37 °C with 5% CO_2_ in Ham’s F-12 K (PM150910, Procell) supplemented with 10% FBS and 1% Penicillin Streptomycin.

#### CCK-8 assay for cell proliferation and cytotoxicity

Three types of lung cells were seeded into 96-well plate at a density of 5 × 10^3^ cells per well at logarithmic growth phase and cultured in 37 °C. Then, the cells were treated initially with different concentrations of compounds (0, 2, 4, 8, 16 and 32 μmol/L) for additional 48 h, with three replica wells each. After that, cell viability was determined according to the instruction of the CCK-8 assay. Next, 10 μL of CCK-8 solution was added to each well of the plate and incubate the plate for 1–4 h in the incubator. The absorbance was measured at 450 nm using a microplate reader (Bio-Tek). The percentage of viable cells was measured using the following formula where three independent experiments were performed: [(A450 _sample_ − A450_blank_)/(A450 _control_ − A450_blank_)] × 100%.

#### Flow cytometry detection for cell apoptosis

H1299 cells were seeded in a 6-well plate at a density of 1 × 10^5^ cells/well and incubated for 24 h. On the following day, the medium was replaced with fresh medium containing **7a** or **7j** (2, 4 and 8 μmol/L) and cells were incubated for an additional 48 h. Cell apoptosis was detected using Annexin V-EGFP/PI apoptosis detection Kit by flow cytometry. Subsequently, 5 µl of EGFR Annexin V and 10 µl of PI was added to the cell suspension, gently vortexed, and incubated at room temperature in the dark for 20 min, then single-cell suspension was prepared followed by flow cytometry (BD Accuri™ C6 Plus).

#### DAPI staining for cell apoptosis

H1299 (1.5 × 10^4^ ~ 2 × 10^4^ /well) cells were seeded in 24-well plates for 24 h, and then treated with **7a** or **7j** at the concentrations of 2,4 and 8 μmol/L for 48 h., the cells were fixed for 30 min at room temperature in 4% paraformaldehyde and washed 3 times in PBS. Finally, every well was stained with DAPI (10 µg/mL, C0065, Solarbio) at room temperature for 30 min, and washed at least three times in PBS. The fluorescence microscope was used to observe the morphological changes of cell nuclei.

#### Wound healing assay

H1299 cells were seeded in a 6-well plate, When the cell confluency reached 95%, a wound line was scratched using a 200 µl pipette tip, and then washed three times, fresh medium containing **7a** or **7j** (2, 4 and 8 μmol/L) was added, and the plate was incubated at 37 °C with 5% CO_2_. All the images were captured at 0 h and 24 h under an inverted microscope, and the quantification of scratches was analysed using Image J.

#### Transwell migration assay

Transwell assay was used to determine the migration ability of H1299 cells. Cells were starved in serum-free RPMI 1640 medium for 24 h, then detached and resuspended in serum-free RPMI 1640 medium. 5 × 10^5^ cells/mL were inoculated into the upper chamber of a 24-well Transwell plate (Corning Inc., United States), with a volume of 100 µl per well. Then, 100 µL serum-free medium containing **7a** or **7j** (2, 4 and 8 μmol/L) was added to the upper chamber and the lower chamber was filled with 700 µL medium containing 20% FBS. After incubation for 24 h, H1299 cells on the upper membrane of the transwell were wiped off. The migrated cells were treated with 4% paraformaldehyde for 20 min, stained with 0.1% crystal violet for 20 min and washed three times with PBS. The number of H1299 cells that migrated to the underside of the membrane was counted under the inverted fluorescence microscope. Three randomly selected areas from each transwell were photographed and calculated using Image J.

#### Colony formation assay

H1299 cells were seeded in six-well plates at a density of 500 cells per well and treated with **7a** or **7j** (1, 2 and 4 μmol/L) for 7 days. Then, cells were per-fixed with 4% paraformaldehyde for 30 min, stained with 0.1% Crystal violet for 15 min, and then washed with pure water. Taking a picture after the plates were air-dry. The clone formations number was counted with Image J.

#### Western blot analysis

Firstly, H1299 cells were treated with different concentrations concentrations of **7a** and **7j** at 4, 8 and 16 μmol/L for 48 h, cells were harvested using RIPA lysate (R0010, Solarbio) and centrifuged at 12,000 rpm for 15 min at 4 °C, and then the concentration of total protein was measured and taken from the supernatant. Protein was separated by 12% SDS–polyacrylamide gel electrophoresis and transferred to nitrocellulose filter membrane. after blocking in 5% milk for 2 h, the membranes were incubated with specific primary antibody at 4 °C overnight. Next day, NC membranes were incubated for 1 h at room temperature with appropriate secondary antibodies, and then Protein bands was visualizing by chemiluminescence detection (ECL kit, Genview).

### In vivo assay

#### Animals

SPF-grade healthy Kunming mice (weighing 20 ± 2 g) were purchased from Henan Skibbes Bio-technology Co., Ltd (Licence No.: SCXK (Yu) 2020–0005). The mice were provided free access to drinking water and adaptive feeding for 7 days.

#### Acute toxicity test

Twenty Kunming mice (half male and half female) were randomly allocate into 4 groups: **7a**-treated group (male), **7a**-treated group (female), control group (male) and control group (female). The control groups were given PBS intragastric administration, while the **7a** groups were given the **7a** solution (400 mg/kg, respectively), once a day for 2 consecutive days. Body weight and appearance were monitored for a total period of 12 days (from the first day after treating with **7a**). The mice were killed by cervical dislocation on the 12th day, and blood samples were collected for the biochemistry test. Then, we measured liver and kidney function indicators such as alanine aminotransferase (ALT), aspartate aminotransferase (AST), serum creatinine (CRE) and blood urea nitrogen (BUN) (Nanjing Jiancheng Bioengineering Institute, China). The organs (brain, heart, liver, spleen, lungs, kidneys stomach) were harvested, and fixed in 10% formaldehyde and paraffin-embedded for histological examination. Finally, these sections were stained with haematoxylin and eosin for light microscopic examination.

### Statistical analyses

We performed statistical analyses using GraphPad Prism 9.5 software these data were analyzed using one-way analysis of variance (ANOVA) followed by Dunnett’s tests. Data were obtained from no fewer than three independent experiments. A* p*-value less than 0.05 was considered statistically significant. A *p*-value more than 0.05 indicated no significant difference.

### Ethical approval

The animal study was reviewed and approved by The Ethics Committee for the Care and Use of Laboratory Animals of Henan University of Science and Technology. All methods are reported in accordance with ARRIVE guidelines and in accordance with the relevant guideline and regulation.

### Supplementary Information

Supplementary Information.

## Data Availability

The data underlying this study are available in the published article and its Supporting Information.
