# Supplementary material for: Discovery of gefitinib-1,2,3-triazole derivatives against lung cancer via inducing apoptosis and inhibiting the colony formation
Source: Sci Rep. 2024 Apr 22;14:9223. doi: 10.1038/s41598-024-60000-1 (PMC11035632; doi:10.1038/s41598-024-60000-1)

**Supplementary Information**

**Discovery of gefitinib-1,2,3-triazole derivatives against lung cancer via inducing apoptosis and inhibiting the colony formation**

En Gao^1,2*^, Ya Wang^1^, Gaolu Fan^3^, Guiqing Xu^1^, Zi-Yuan Wu^4^, Zi-Jun Liu^4^, Jian-Cheng Liu^4^, Long-Fei Mao^4*^, Xixi Hou^5^, Shouhu Li^6^

^1^School of Chemistry and Chemical Engineering, Henan Normal University, Xinxiang, 453000, China

^2^State Key Laboratory of Quality Research in Chinese Medicine/Macau Institute for Applied Research in Medicine and Health, Macau University of Science and Technology, Macau, China

^3^Department of Pharmacy, Luoyang third people’ hospital, Luoyang 471000, China

^4^College of Basic Medicine and Forensic Medicine, Henan University of Science and Technology, 263 Kaiyuan Road, Luoyang 471003, China

^5^Department of Pharmacy, The First Affiliated Hospital, and College of Clinical Medicine of Henan University of Science and Technology, Luoyang, 471003, China

^6^School of Pharmacy, Xinxiang University, Xinxiang, Henan 453000, China

**Table of Content**

[The detail data of NMR and HRMS spectrums. 1](#_Toc155800128)

[Figure S1-1. ^1^H NMR spectrum (400MHz, DMSO-*d*_6_) of compound 7a 8](#_Toc155800129)

[Figure S1-2. ^13^C NMR spectrum (101MHz, DMSO-*d*_6_) of compound 7a 9](#_Toc155800130)

[Figure S1-3. HR MS of compound 7a 9](#_Toc155800131)

[Figure S2-1. ^1^H NMR spectrum (400MHz, Chloroform-d) of compound 7b 10](#_Toc155800132)

[Figure S2-2. ^13^C NMR spectrum (101MHz, DMSO-*d*_6_) of compound 7b 11](#_Toc155800133)

[Figure S2-3. HR MS of compound 7b 11](#_Toc155800134)

[Figure S3-1. ^1^H NMR spectrum (400MHz, DMSO-*d*_6_) of compound 7c 12](#_Toc155800135)

[Figure S3-2. ^13^C NMR spectrum (101MHz, DMSO-*d*_6_) of compound 7c 13](#_Toc155800136)

[Figure S3-3. HR MS of compound 7c 13](#_Toc155800137)

[Figure S4-1. ^1^H NMR spectrum (400MHz, DMSO-*d*_6_) of compound 7d 14](#_Toc155800138)

[Figure S4-2. ^13^C NMR spectrum (101MHz, DMSO-*d*_6_) of compound 7d 15](#_Toc155800139)

[Figure S4-3. HR MS of compound 7d 15](#_Toc155800140)

[Figure S5-1. ^1^H NMR spectrum (400MHz, DMSO-*d*_6_) of compound 7e 16](#_Toc155800141)

[Figure S5-2. ^13^C NMR spectrum (101MHz, DMSO-*d*_6_) of compound 7e 17](#_Toc155800142)

[Figure S5-3. HR MS of compound 7e 17](#_Toc155800143)

[Figure S6-1. ^1^H NMR spectrum (400MHz, DMSO-*d*_6_) of compound 7f 18](#_Toc155800144)

[Figure S6-2. ^13^C NMR spectrum (101MHz, DMSO-*d*_6_) of compound 7f 19](#_Toc155800145)

[Figure S6-3. HR MS of compound 7f 19](#_Toc155800146)

[Figure S7-1. ^1^H NMR spectrum (400MHz, DMSO-*d*_6_) of compound 7g 20](#_Toc155800147)

[Figure S7-2. ^13^C NMR spectrum (101MHz, DMSO-*d*_6_) of compound 7g 21](#_Toc155800148)

[Figure S7-3. HR MS of compound 7g 21](#_Toc155800149)

[Figure S8-1. ^1^H NMR spectrum (400MHz, DMSO-*d*_6_) of compound 7h 22](#_Toc155800150)

[Figure S8-2. ^13^C NMR spectrum (101MHz, DMSO-*d*_6_) of compound 7h 23](#_Toc155800151)

[Figure S8-3. HR MS of compound 7h 23](#_Toc155800152)

[Figure S9-1. ^1^H NMR spectrum (400MHz, DMSO-*d*_6_) of compound 7i 24](#_Toc155800153)

[Figure S9-2. ^13^C NMR spectrum (101MHz, DMSO-*d*_6_) of compound 7i 25](#_Toc155800154)

[Figure S9-3. HR MS of compound 7i 25](#_Toc155800155)

[Figure S10-1. ^1^H NMR spectrum (400MHz, DMSO-*d*_6_) of compound 7j 26](#_Toc155800156)

[Figure S10-2. ^13^C NMR spectrum (101MHz, DMSO-*d*_6_) of compound 7j 27](#_Toc155800157)

[Figure S10-3. HR MS of compound 7j 27](#_Toc155800158)

[Figure S11-1. ^1^H NMR spectrum (400MHz, DMSO-*d*_6_) of compound 7k 28](#_Toc155800159)

[Figure S11-2. ^13^C NMR spectrum (101MHz, DMSO-*d*_6_) of compound 7k 29](#_Toc155800160)

[Figure S11-3. HR MS of compound 7k 29](#_Toc155800161)

[Figure S12-1. ^1^H NMR spectrum (400MHz, DMSO-*d*_6_) of compound 7l 30](#_Toc155800162)

[Figure S12-2. ^13^C NMR spectrum (101MHz, DMSO-*d*_6_) of compound 7l 31](#_Toc155800163)

[Figure S12-3. HR MS of compound 7l 31](#_Toc155800164)

[Figure S13-1. ^1^H NMR spectrum (400MHz, DMSO-*d*_6_) of compound 7m 32](#_Toc155800165)

[Figure S13-2. ^13^C NMR spectrum (101MHz, DMSO-*d*_6_) of compound 7m 33](#_Toc155800166)

[Figure S13-3. HR MS of compound 7m 33](#_Toc155800167)

[Figure S14-1. ^1^H NMR spectrum (400MHz, DMSO-*d*_6_) of compound 7n 34](#_Toc155800168)

[Figure S14-2. ^13^C NMR spectrum (101MHz, DMSO-*d*_6_) of compound 7n 35](#_Toc155800169)

[Figure S14-3. HR MS of compound 7n 35](#_Toc155800170)

[Figure S15-1. ^1^H NMR spectrum (400MHz, DMSO-*d*_6_ of compound 7o 36](#_Toc155800171)

[Figure S15-2. ^13^C NMR spectrum (101MHz, DMSO-*d*_6_) of compound 7o 37](#_Toc155800172)

[Figure S15-3. HR MS of compound 7o 37](#_Toc155800173)

[Figure S16-1. ^1^H NMR spectrum (400MHz, DMSO-*d*_6_) of compound 7p 38](#_Toc155800174)

[Figure S16-2. ^13^C NMR spectrum (101MHz, DMSO-*d*_6_) of compound 7p 39](#_Toc155800175)

[Figure S16-3. HR MS of compound 7p 39](#_Toc155800176)

[Figure S17-1. ^1^H NMR spectrum (400MHz, DMSO-*d*_6_) of compound 7q 40](#_Toc155800177)

[Figure S17-2. ^13^C NMR spectrum (101MHz, DMSO-*d*_6_) of compound 7q 41](#_Toc155800178)

[Figure S17-3. HR MS of compound 7q 41](#_Toc155800179)

[Figure S18-1. ^1^H NMR spectrum (400MHz, DMSO-*d*_6_) of compound 7r 42](#_Toc155800180)

[Figure S18-2. ^13^C NMR spectrum (101MHz, DMSO-*d*_6_) of compound 7r 43](#_Toc155800181)

[Figure S18-3. HR MS of compound 7r 43](#_Toc155800182)

[Figure S19-1. ^1^H NMR spectrum (400MHz, DMSO-*d*_6_) of compound 7s 44](#_Toc155800183)

[Figure S19-2. ^13^C NMR spectrum (101MHz, DMSO-*d*_6_) of compound 7s 45](#_Toc155800184)

[Figure S19-3. HR MS of compound 7s 45](#_Toc155800185)

[Figure S20-1. ^1^H NMR spectrum (400MHz, DMSO-*d*_6_) of compound 7t 46](#_Toc155800186)

[Figure S20-2. ^13^C NMR spectrum (101MHz, DMSO-*d*_6_) of compound 7t 47](#_Toc155800187)

[Figure S20-3. HR MS of compound 7t 47](#_Toc155800188)

# The detail data of NMR and HRMS spectrums.

**N-(3-(1-(2-bromo-5-fluorobenzyl)-1H-1,2,3-triazol-4-yl)phenyl)-7-methoxy-6-(3-morpholinopropoxy)quinazolin-4-amine** **(7a):** Yellow solid, yield 85%, ^1^H NMR (400 MHz, DMSO-*d*_6_) *δ* 9.63 (s, 1H), 8.64 (s, 1H), 8.26 (s, 1H), 8.13 – 8.06 (m, 1H), 7.90 (d, *J* = 8.0 Hz, 1H), 7.77-7.75(m, 1H), 7.58 (d, *J* = 7.6 Hz, 1H), 7.45 (s, 1H), 7.30 – 7.15 (m, 2H), 5.75 (s, 2H), 4.25 – 4.15 (m, 2H), 3.98 (s, 3H), 3.61 – 3.53 (m, 4H), 2.44 – 2.37 (m, 3H), 2.09 – 1.92 (m, 2H). ^13^C NMR (101 MHz, DMSO) *δ* 163.1, 160.7, 154.6, 148.9, 147.0, 140.5, 137.4(d, *J* = 7.6 Hz), 135.2(d, *J* = 8.0 Hz), 131.3, 129.5,128.6, 128.5, 122.6, 120.9, 119.4, 118.4, 118.2, 118.1,118.0, 103.667.6, 66.6, 66.5, 56.4, 55.4, 53.9, 53.4, 53.1, 26.3. HR MS (ESI) m/z: calcd for C_37_H_35_N_9_O_7_ [M + H]^+^ 718.2732, found 728.2731.

**N-(3-(1-(4-bromo-2-fluorobenzyl)-1H-1,2,3-triazol-4-yl)phenyl)-7-methoxy-6-(3-morpholinopropoxy)quinazolin-4-amine** **(7b):** Yellow solid, yield 81%, ^1^H NMR (400 MHz, Chloroform-*d*) *δ* 9.60 (s, 1H), 8.64 (s, 1H), 8.26 (s, 1H), 8.02 (s, 1H), 7.91 (d, *J* = 8.0 Hz, 1H), 7.65 (d, J = 9.6 Hz, 1H), 7.56 (d, *J* = 7.6 Hz, 1H), 7.54 – 7.31 (m, 3H), 5.71 (s, 2H), 4.21 (t, *J* = 6.3 Hz, 2H), 3.97 (s, 3H), 3.64 – 3.52 (m, 4H), 2.43 – 2.40 (m, 3H), 2.01 (t, *J* = 6.8 Hz, 2H). ^13^C NMR (101 MHz, DMSO) *δ* 164.0, 161.8, 159.3, 154.8, 148.8, 147.0, 140.5, 132.8(d, *J* = 3.8 Hz), 131.2, 129.5, 128.6 (d, *J* = 3.6 Hz), 122.9, 122.8, 122.7, 122.5, 122.3, 120.8, 119.7, 119.5, 119.3, 103.4, 67.6, 66.6, 56.4, 55.4, 53.9, 47.2, 47.1, 26.3. HR MS (ESI) m/z: calcd for C_37_H_35_N_9_O_7_ [M + H]^+^ 718.2732, found 728.2731.

**N-(3-(1-(4-chloro-3-fluorobenzyl)-1H-1,2,3-triazol-4-yl)phenyl)-7-methoxy-6-(3-morpholinopropoxy)quinazolin-4-amine (7c):** Yellow solid, yield 79%, ^1^H NMR (400 MHz, DMSO-*d*_6_) *δ* 9.60 (s, 1H), 8.61 (s, 1H), 8.25 (s, 1H), 8.02 - 7.90 (m, 2H), 7.65 – 7.16 (m, 6H), 5.75 (s, 2H), 4.21 (t, *J* = 6.5 Hz, 2H), 3.97 (s, 3H), 3.71 – 3.52 (m, 4H), 2.47 – 2.25 (m, 4H), 2.09 – 1.89 (m, 2H). ^13^C NMR (101 MHz, DMSO) *δ* 163.6, 161.1,158.4, 154.7, 148.8, 146.9, 140.5, 134.4 (d, *J* = 10.5 Hz), 133.1 (d, *J* = 9.4 Hz), 131.3, 130.1, 129.5, 122.5, 122.4, 120.9, 119.4, 117.7, 117.4, 115.6, 115.3,103.4, 93.3, 67.6, 66.6, 56.4, 55.4, 53.9, 50.7, 26.3. HR MS (ESI) m/z: calcd for C_31_H_31_ClFN_7_O_3_ [M + H]^+^ 605.2234, found 605.2234.

**N-(3-(1-(2-chloro-6-fluorobenzyl)-1H-1,2,3-triazol-4-yl)phenyl)-7-methoxy-6-(3-morpholinopropoxy)quinazolin-4-amine (7d):** Yellow solid, yield 75%, ^1^H NMR (400 MHz, DMSO-*d*_6_) *δ* 9.62 (s, 1H), 8.62 (s, 1H), 8.23 (s, 1H), 8.12 (s, 1H), 7.90 (d, *J* = 8.1 Hz, 1H), 7.68 – 7.13 (m, 6H), 5.78 (s, 2H), 4.21 (t, *J* = 6.4 Hz, 2H), 3.98 (s, 3H), 3.64 – 3.53 (m, 4H), 2.47 – 2.35 (m, 4H), 2.01 (t, *J* = 6.9 Hz, 2H). ^13^C NMR (101 MHz, DMSO) *δ* 163.1, 160.6,156.3, 154.6, 148.9, 146.7, 140.5, 135.5 (d, *J* = 4.8 Hz), 132.4 (d, *J* = 9.8 Hz), 132.3, 131.2, 129.4, 129.1, 126.4 (d, *J* = 3.4 Hz), 122.6, 122.3, 121.4, 121.2, 121.0, 119.4, 115.6, 115.4, 67.6, 66.6, 56.4, 55.4, 53.8, 45.1, 26.3. HR MS (ESI) m/z: calcd for C_31_H_31_ClFN_7_O_3_ [M + H]^+^ 604.2234, found 605.2232.

**7-methoxy-6-(3-morpholinopropoxy)-N-(3-(1-(2-nitrobenzyl)-1H-1,2,3-triazol-4-yl)phenyl)quinazolin-4-amine (7e):** Yellow solid, yield 71%, ^1^H NMR (400 MHz, DMSO-*d*_6_) *δ* 9.62 (s, 1H), 8.64 (s, 1H), 8.28 (s, 1H), 8.18 (d, *J* = 8.1 Hz, 1H), 8.03 (s, 1H), 7.94 – 7.86 (m, 1H), 7.82 – 7.74 (m, 1H), 7.71 – 7.62 (m, 1H), 7.61 – 7.56 (m, 1H), 7.60 – 7.54 (m, 1H), 7.49 – 7.43 (m, 1H), 7.19 (d, *J* = 7.8 Hz, 1H), 6.04 (s, 2H), 4.20 (t, *J* = 6.3 Hz, 2H), 3.97 (s, 3H), 3.64 – 3.52 (m, 4H), 2.45 – 2.29 (m, 4H), 2.10 – 1.95 (m, 2H). ^13^C NMR (101 MHz, DMSO) *δ* 156.4, 154.7, 148.8, 148.1, 147.0, 140.6, 134.9, 131.2, 131.1, 130.7, 130.2, 129.5, 125.6, 122.9, 122.5, 120.9, 119.4, 103.4, 67.6, 66.6, 56.4, 55.4, 55.3, 53.9, 50.7, 26.3. HR MS (ESI) m/z: calcd for C_31_H_33_N_8_O_5_ [M + H]^+^ 597.2568, found 597.2567.

**7-methoxy-6-(3-morpholinopropoxy)-N-(3-(1-(4-nitrobenzyl)-1H-1,2,3-triazol-4-yl)phenyl)quinazolin-4-amine (7f):** Yellow solid, yield 55%, ^1^H NMR (400 MHz, DMSO-*d*_6_) *δ* 9.63 (s, 1H), 8.72 (s, 1H), 8.34 – 8.19 (m, 3H), 8.02 – 7.80 (m, 2H), 7.69 – 7.52 (m, 3H), 7.47 (t, *J* = 7.7 Hz, 2H), 7.28 (s, 1H), 5.86 (s, 2H), 4.70 (s, 1H), 4.22 (t, *J* = 7.7 Hz, 4H), 3.95 (s, 3H), 3.63 – 3.52 (m, 4H), 2.47 – 2.37 (m, 4H), 2.08 – 1.94 (m, 2H). ^13^C NMR (101 MHz, DMSO) *δ* 156.8, 154.9, 148.8, 147.8, 147.2, 143.9, 140.5, 135.9, 131.9, 131.5, 131.2, 129.5, 129.4, 124.4, 122.6, 122.5, 120.9, 119.4, 103.2, 67.6, 66.5, 56.3, 55.4, 53.8, 52.6, 49.4, 26.2 HR MS (ESI) m/z: calcd for C_31_H_33_N_8_O_5_ [M + H]^+^ 597.2568, found 597.2567.

**N-(3-(1-(2-fluorobenzyl)-1H-1,2,3-triazol-4-yl)phenyl)-7-methoxy-6-(3-morpholinopropoxy)quinazolin-4-amine (7g):** Yellow solid, yield 50%, ^1^H NMR (400 MHz, DMSO-*d*_6_) *δ* 9.61 (s, 1H), 8.64 (s, 1H), 8.25 (s, 1H), 7.93 – 7.86 (m, 1H), 7.61 – 7.53 (m, 1H), 7.48 – 7.37 (m, 3H), 7.32 – 7.21 (m, 2H), 5.73 (s, 2H), 4.21 (d, *J* = 6.5 Hz, 2H), 3.99 (s, 3H), 3.61 – 3.56 (m, 4H), 2.48 – 2.39 (m, 4H), 2.06 – 1.96 (m, 2H). ^13^C NMR (101 MHz, DMSO) *δ* 161.8, 159.4, 154.6, 148.9, 147.0, 140.5, 131.3, 131.2 (d, J = 3.2 Hz), 129.5, 125.4(d, J = 3.4 Hz), 125.3, 123.3, 123.2, 122.4, 122.2, 120.8, 119.3, 116.2, 116.0, 80.5, 73.7, 67.6, 66.5, 59.9, 56.4, 55.4, 53.8, 47.6, 26.2~~.~~ HR MS (ESI) m/z: calcd for C_31_H_32_FN_7_O_3_ [M + H]^+^ 570.2623, found 570.2635.

**7-methoxy-6-(3-morpholinopropoxy)-N-(3-(1-(4-(trifluoromethyl)benzyl)-1H-1,2,3-triazol-4-yl)phenyl)quinazolin-4-amine (7h):** Yellow solid, yield 60%, ^1^H NMR (400 MHz, DMSO-*d*_6_) *δ* 9.61 (s, 1H), 8.71 (s, 1H), 8.28 (s, 1H), 8.03 (s, 1H), 7.90 (d, *J* = 8.0 Hz, 1H), 7.78 (d, *J* = 7.9 Hz, 2H), 7.62 – 7.52 (m, 3H), 7.50 – 7.36 (m, 1H), 5.80 (s, 2H), 4.21 (t, J = 4.0 Hz, 2H), 3.97 (s, 3H), 3.61 – 3.55 (m,4H), 2.47 – 2.30 (m, 4H), 2.13 – 1.88 (m, 2H). ^13^C NMR (101 MHz, DMSO) *δ* 156.5, 154.7,152.7, 149.4, 148.8, 147.2, 141.1, 140.6, 131.3, 129.5, 129.1, 129.0, 126.2 (d, J = 3.8 Hz), 126.1, 123.2, 122.8,122.4, 120.8, 119.3, 103.4, 76.9, 67.6, 66.6, 56.4, 55.4, 53.9, 52.9, 26.3. HR MS (ESI) m/z: calcd for C_32_H_32_F_3_N_7_O_3_ [M + H]^+^ 620.2591, found 620.2604.

**N-(3-(1-(4-iodobenzyl)-1H-1,2,3-triazol-4-yl)phenyl)-7-methoxy-6-(3-morpholinopropoxy)quinazolin-4-amine (7i):** Yellow solid, yield 65%, ^1^H NMR (400 MHz, DMSO-*d*_6_) *δ* 9.59 (s, 1H), 8.65 (s, 1H), 8.26 (s, 1H), 7.98 – 7.84 (m, 2H), 7.80 – 7.69 (m, 2H), 7.61 – 7.52 (m, 1H), 7.47 – 7.40 (m, 1H), 7.26 – 7.04 (m, 2H), 5.63 (s, 2H), 4.22 (t, *J* = 4 Hz, 4H), 3.96 (s, 3H), 3.66 – 3.59 (m, 4H), 2.65 – 2.55 (m, 2H), 2.26 – 1.98 (m, 2H). ^13^C NMR (101 MHz, DMSO) *δ* 156.7, 154.8, 148.7, 148.6, 147.2, 147.1, 140.5, 138.1, 136.2, 131.3, 130.7, 129.5, 124.6, 122.4, 122.2, 120.8, 119.3,

103.3, 95.0, 67.5, 66.3, 56.3, 55.3, 53.7, 52.9, 31.8, 26.0~~.~~ HR MS (ESI) m/z: calcd for C_31_H_32_IN_7_O_3_ [M + H]^+^ 678.1684, found 678.2441.

**N-(3-(1-(3-chlorobenzyl)-1H-1,2,3-triazol-4-yl)phenyl)-7-methoxy-6-(3-morpholinopropoxy)quinazolin-4-amine (7j):** Yellow solid, yield 67%, ^1^H NMR (400 MHz, DMSO-*d*_6_) *δ* 9.60 (s, 1H), 8.70 (s, 1H), 8.27 (s, 1H), 8.03 – 7.84 (m, 2H), 7.63 – 7.53 (m, 1H), 7.52 – 7.41 (m, 5H), 7.34 (s, 1H), 5.69 (s, 2H), 4.49 (s, 1H), 4.22 (d, *J* = 6.4 Hz, 2H), 3.96 (s, 3H), 3.67 – 3.53 (m, 4H), 2.44 – 2.30 (m, 3H), 2.10 – 1.91 (m, 2H). ^13^C NMR (101 MHz, DMSO) *δ* 154.8, 148.8, 147.2, 140.6, 138.8, 138.7, 133.8, 133.7, 131.3, 131.2, 131.1, 129.5, 128.7, 128.5, 128.4, 127.5, 127.2, 122.4, 122.3, 120.8, 119.3, 67.6, 66.6, 56.3, 55.4, 53.9, 53.2, 52.8, 26.3. HR MS (ESI) m/z: calcd for C_31_H_32_ClN_7_O_3_ [M + H]^+^ 586.3005, found 586.3010.

**N-(3-(1-(4-chlorobenzyl)-1H-1,2,3-triazol-4-yl)phenyl)-7-methoxy-6-(3-morpholinopropoxy)quinazolin-4-amine (7k):** Yellow solid, yield 70%, ^1^H NMR (400 MHz, DMSO-*d*_6_) *δ* 9.60 (s, 1H), 8.66 (s, 1H), 8.26 (s, 1H), 8.01 – 7.81 (m, 2H), 7.61 – 7.29 (m, 7H), 5.68 (s, 2H), 4.21 (d, *J* = 6.4 Hz, 2H), 3.95 (s, 3H), 3.63 – 3.56 (m, 5H), 2.45 – 2.30 (m, 3H), 2.07 – 1.95 (m, 2H). ^13^C NMR (101 MHz, DMSO) *δ* 156.6, 154.7, 149.4, 148.8, 147.2, 140.5, 135.5, 138.1, 135.4, 133.4, 131.3, 130.4, 129.5, 129.3, 122.4, 122.2, 120.8, 119.3, 103.4, 72.9, 67.6, 66.6, 56.3, 55.4, 53.9, 52.7, 26.3. HR MS (ESI) m/z: calcd for C_31_H_32_ClN_7_O_3_ [M + H]^+^ 586.3045, found 586.3041.

**7-methoxy-N-(3-(1-(2-methylbenzyl)-1H-1,2,3-triazol-4-yl)phenyl)-6-(3-morpholinopropoxy)quinazolin-4-amine (7l):** Yellow solid, yield 65%, ^1^H NMR (400 MHz, DMSO-*d*_6_) *δ* 9.60 (s, 1H), 8.56 (s, 1H), 8.25 (s, 1H), 8.06 (s, 1H), 7.90 (d, *J* = 8.0 Hz, 1H), 7.60 – 7.53 (m, 1H), 7.50 – 7.39 (m, 1H), 7.31 – 7.10 (m, 4H), 5.67 (s, 2H), 4.21 (t, *J* = 6.1 Hz, 2H), 3.97 (s, 3H), 3.67 – 3.52 (m, 4H), 2.41 – 2.29 (m, 6H), 2.08 – 1.95 (m, 2H). ^13^C NMR (101 MHz, DMSO) *δ* 154.7, 148.8, 147.0, 140.5, 136.8, 134.6, 131.4, 130.9, 129.5, 129.2, 128.8, 126.8, 122.4, 122.1, 122.1, 120.8, 119.4, 111.6, 103.4, 67.6, 66.6, 60.2, 56.4, 55.4, 53.9, 51.6, 26.3, 21.2, 19.2, 14.6~~.~~ HR MS (ESI) m/z: calcd for C_32_H_35_N_7_O_3_ [M + H]^+^ 566.3589, found 566.3591.

**N-(3-(1-(3,5-dimethylbenzyl)-1H-1,2,3-triazol-4-yl)phenyl)-7-methoxy-6-(3-morpholinopropoxy)quinazolin-4-amine (7m):** Yellow solid, yield 64%, ^1^H NMR (400 MHz, DMSO-*d*_6_) *δ* 9.60 (s, 1H), 8.63 (s, 1H), 8.26 (s, 1H), 8.06 – 8.00 (m, 1H), 7.89 (d, *J* = 8.0 Hz, 1H), 7.56 (d, *J* = 7.6 Hz, 1H), 7.49 – 7.41 (m, 1H), 6.98 (s, 3H), 5.56 (s, 2H), 4.21 (t, *J* = 6.3 Hz, 2H), 3.96 (s, 3H), 3.65 – 3.49 (m, 4H), 2.46 – 2.35 (m, 4H), 2.08 – 1.93 (m, 2H). ^13^C NMR (101 MHz, DMSO) *δ* 156.6, 154.7, 148.8, 147.1,144.9, 143.5, 141.8, 140.5, 138.4, 136.3, 131.4, 130.0, 129.5, 126.2, 122.4, 122.0, 120.8, 119.3,103.4, 82.6, 67.6, 66.6, 59.9, 56.4, 55.4, 53.9, 53.5, 26.3, 21.3. HR MS (ESI) m/z: calcd for C_33_H_37_N_7_O_3_ [M + H]^+^ 580.3780, found 580.3782.

**4-fluoro-2-((4-(3-((7-methoxy-6-(3-morpholinopropoxy)quinazolin-4-yl)amino)phenyl)-1H-1,2,3-triazol-1-yl)methyl)benzonitrile (7n):** Yellow solid, yield 58%, ^1^H NMR (400 MHz, DMSO-*d*_6_) *δ* 9.61 (s, 1H), 8.70 (s, 1H), 8.27 (s, 1H), 8.12 – 8.00 (m, 2H), 7.94 – 7.84 (m, 1H), 7.61 – 7.30 (m, 5H), 5.89 (s, 2H), 4.21 (t, *J* = 6.4 Hz, 2H), 3.97 (s, 3H), 3.62 – 3.54 (m, 4H), 2.44 – 2.38 (m, 4H), 2.01 (t, *J* = 6.8 Hz, 2H). ^13^C NMR (101 MHz, DMSO) *δ* 166.2, 163.7, 156.5, 154.8, 148.8, 147.1, 142.6(d, J = 9.3 Hz), 140.6, 137.0, 136.9, 131.2, 129.5, 122.7,122.6, 120.9, 119.4, 118.0, 117.7, 117.5, 117.2, 116.8, 108.5(d, J = 3.5 Hz), 103.4, 67.6, 66.6, 56.4, 55.4, 53.9, 51.4, 26.3. HR MS (ESI) m/z: calcd for C_32_H_31_FN_8_O_3_ [M + H]^+^ 595.3353, found 595.3357.

**N-(3-(1-(3,5-dimethoxybenzyl)-1H-1,2,3-triazol-4-yl)phenyl)-7-methoxy-6-(3-morpholinopropoxy)quinazolin-4-amine (7o):** Yellow solid, yield 55%, ^1^H NMR (400 MHz, DMSO-*d*_6_) *δ* 9.61 (s, 1H), 8.65 (s, 1H), 8.32 – 8.00 (m, 2H), 7.90 (s, 1H), 7.61 – 7.15 (m, 4H), 6.59 – 6.42 (m, 3H), 5.57 (s, 2H), 4.21 (s, 2H), 3.98 (s, 3H), 3.73 (s, 6H), 3.63 – 3.51 (m, 4H), 2.47 – 2.33 (m, 4H), 2.05 – 1.91 (m, 2H). ^13^C NMR (101 MHz, DMSO) *δ* 161.2, 158.4, 158.2, 154.7, 148.9, 147.1, 140.5, 138.5, 131.4, 129.5, 122.4, 122.1, 120.8, 119.3, 115.2, 115.0, 106.6, 103.6, 100.1, 67.6, 66.6, 56.4, 55.7, 55.4, 53.9, 53.5, 51.2, 26.3. HR MS (ESI) m/z: calcd for C_33_H_37_N_7_O_5_ [M + H]^+^ 612.3712, found 612.3714

**N-(3-(1-(3-iodobenzyl)-1H-1,2,3-triazol-4-yl)phenyl)-7-methoxy-6-(3-morpholinopropoxy)quinazolin-4-amine (7p):** Yellow solid, yield 52%, ^1^H NMR (400 MHz, DMSO-*d*_6_) *δ* 9.60 (s, 1H), 8.68 (s, 1H), 8.27 (s, 1H), 7.98 – 7.85 (m, 2H), 7.79 (s, 1H), 7.73 (d, *J* = 7.9 Hz, 1H), 7.56 (d, *J* = 7.7 Hz, 1H), 7.46 (t, *J* = 7.9 Hz, 1H), 7.38 (d, *J* = 7.7 Hz, 1H), 7.25 – 7.15 (m, 1H), 5.65 (s, 2H), 4.21 (t, *J* = 6.3 Hz, 2H), 3.95 (s, 3H), 3.62 – 3.55 (m, 4H), 2.46 – 2.39 (m, 4H), 2.01 (t, *J* = 6.7 Hz, 2H). ^13^C NMR (101 MHz, DMSO) *δ* 156.7, 154.8, 148.8, 147.2, 140.6, 138.9, 137.4, 137.0, 135.8, 131.5, 131.3, 129.5, 127.9, 122.4, 122.2, 120.8, 119.3, 103.3, 95.6, 81.1, 71.0, 69.6, 67.6, 66.6, 56.3, 55.4, 53.9, 52.6, 26.3. HR MS (ESI) m/z: calcd for C_31_H_32_IN_7_O_3_ [M +H]^+^ 678.2610, found 678.2611.

**N-(3-(1-(2-fluorophenyl)-1H-1,2,3-triazol-4-yl)phenyl)-7-methoxy-6-(3-morpholinopropoxy)quinazolin-4-amine (7q):** Yellow solid, yield 47%, ^1^H NMR (400 MHz, DMSO-*d*_6_) *δ* 9.68 (s, 1H), 9.11 (s, 1H), 8.37 (s, 2H), 8.01 – 7.84 (m, 2H), 7.77 – 7.57 (m, 3H), 7.53 – 7.40 (m, 2H), 4.33 – 4.14 (m, 2H), 4.05 (s, 3H), 3.65 – 3.52 (m, 4H), 2.46 – 2.33 (m, 4H), 2.07 – 1.93 (m, 2H), 1.29 – 1.13 (m, 2H). ^13^C NMR (101 MHz, DMSO) *δ* 173.6, 172.7, 170.7, 159.2, 158.9,157.9, 156.7, 155.6, 147.3 (d, *J* = 3.2 Hz), 140.7 140.7 (d, *J* = 3.4 Hz), 131.9, 130.8, 129.7, 126.5, 126.1,125.3, 123.4, 122.9, 121.1, 119.6, 117.8, 117.8, 67.7, 66.6, 55.4, 53.9, 26.4. HR MS (ESI) m/z: calcd for C_30_H_30_FN_7_O_3_ [M + H]^+^ 556.3220, found 556.3223.

**7-methoxy-N-(3-(1-(3-methoxyphenyl)-1H-1,2,3-triazol-4-yl)phenyl)-6-(3-morpholinopropoxy)quinazolin-4-amine (7r):** Yellow solid, yield 48%, ^1^H NMR (400 MHz, DMSO-*d*_6_) *δ* 9.67 (s, 1H), 9.35 (s, 1H), 8.36 (s, 1H), 8.10 – 7.87 (m, 2H), 7.71 – 7.64 (m, 1H), 7.61 – 7.46 (m, 5H), 7.15 – 6.98 (m, 1H), 4.22 (t, *J* = 6.3 Hz, 2H), 3.97(s, 3H), 3.89 (s, 3H), 3.59 (t, *J* = 4.5 Hz, 4H), 3.51 (s, 1H), 2.44 – 2.34 (m, 4H), 2.10 – 1.94 (m, 2H).^13^C NMR (101 MHz, DMSO) *δ* 160.7, 154.7, 149.0, 148.8, 147.7, 140.6, 138.1, 131.4, 131.0, 129.6, 122.9, 121.0, 120.2, 119.6, 114.9, 112.4, 106.1,103.4, 95.5, 70.3, 67.6, 66.6, 56.4, 56.1, 55.4, 53.9, 29.5, 26.3. HR MS (ESI) m/z: calcd for C_31_H_33_N_7_O_4_ [M + H]^+^ 568.3445, found 568.3448.

**7-methoxy-6-(3-morpholinopropoxy)-N-(3-(1-(3-nitrophenyl)-1H-1,2,3-triazol-4-yl)phenyl)quinazolin-4-amine (7s):** Yellow solid, yield 52%, ^1^H NMR (400 MHz, DMSO-*d*_6_) *δ* 9.65 (s, 1H), 9.59 (s, 1H), 8.82 (d, *J* = 2.3 Hz, 1H), 8.53 – 8.46 (m, 2H), 8.43 – 8.29 (m, 2H), 8.02 – 7.87 (m, 3H), 7.68 (d, J = 7.6 Hz, 1H), 7.54 (t, *J* = 7.9 Hz, 1H), 7.21 (s, 1H), 4.30-4.15 (m, 1H), 3.95 (s, 3H), 3.59 (t, *J* = 4.5 Hz, 4H), 2.50 – 2.48 (m, 1H), 2.46 – 2.32 (m, 4H), 2.06 – 1.93 (m, 2H). ^13^C NMR (101 MHz, DMSO) *δ* 156.9, 154.9, 153.3, 149.1, 148.8, 148.2, 140.7, 137.7, 135.9, 132.1, 130.6, 129.7, 126.4, 125.3, 123.6, 123.0, 121.0, 120.6, 119.6, 115.1, 107.8, 103.2, 67.6, 66.6, 56.3, 55.5, 53.9, 26.3. HR MS (ESI) m/z: calcd for C_30_H_30_N_8_O_5_ [M + H]^+^ 583.3220, found 583.3222.

**7-methoxy-N-(3-(1-(2-methoxy-4-nitrophenyl)-1H-1,2,3-triazol-4-yl)phenyl)-6-(3-morpholinopropoxy)quinazolin-4-amine (7t)**: Yellow solid, yield 55%, ^1^H NMR (400 MHz, DMSO-*d*_6_) *δ* 9.65 (s, 1H), 9.11 (s, 1H), 8.37 (s, 1H), 8.17 – 7.99 (m, 3H), 7.95 (d, J = 7.9 Hz, 2H), 7.70 (d, J = 7.9 Hz, 2H), 7.60 – 7.40 (m, 1H), 4.39 – 4.13 (m, 2H), 4.08 (s, 3H), 3.96 (s, 3H), 3.62 – 3.50 (m, 4H), 2.45 – 2.34 (m, 4H), 2.11 – 1.84 (m, 2H). ^13^C NMR (101 MHz, DMSO) *δ* 156.7, 152.1, 148.8, 148.7, 147.2, 140.6, 131.0, 130.7, 129.6, 126.5, 123.8, 122.9, 121.2, 119.6, 116.8, 108.8, 103.3, 67.6, 66.6, 57.7, 56.4, 55.4, 53.9, 26.3. HR MS (ESI) m/z: calcd for C_31_H_32_N_8_O_6_ [M + H]+ 613.3376, found 613.3378.

# Figure S1-1. ^1^H NMR spectrum (400MHz, DMSO-*d*_6_) of compound 7a

# Figure S1-2. ^13^C NMR spectrum (101MHz, DMSO-*d*_6_) of compound 7a

# Figure S1-3. HR MS of compound 7a


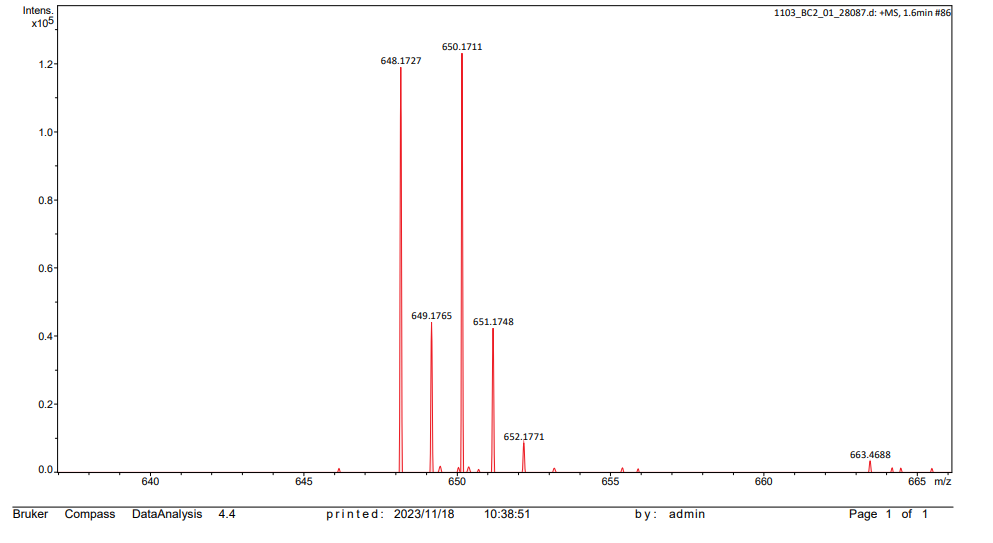


# Figure S2-1. ^1^H NMR spectrum (400MHz, Chloroform-d) of compound 7b

# Figure S2-2. ^13^C NMR spectrum (101MHz, DMSO-*d*_6_) of compound 7b

# Figure S2-3. HR MS of compound 7b


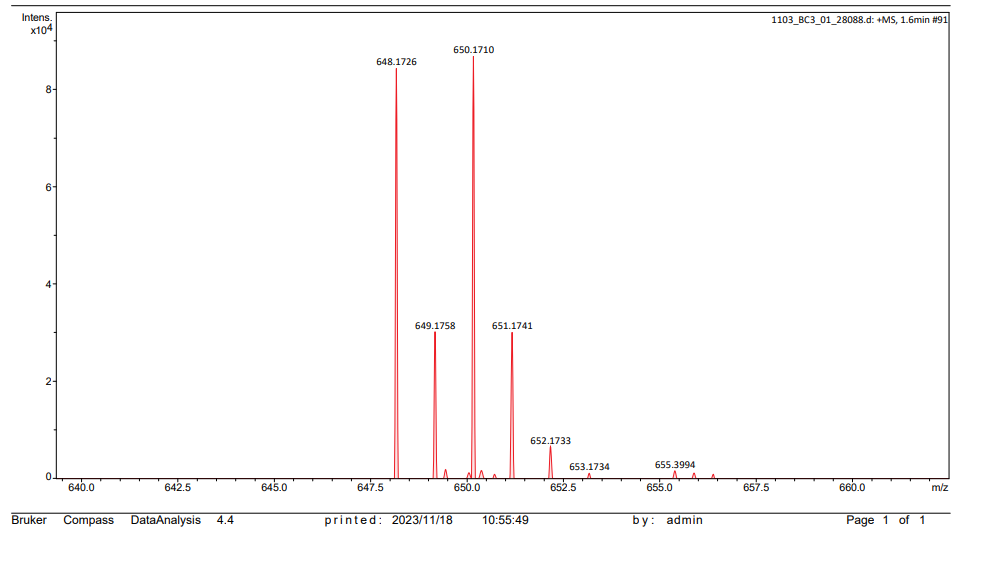


# Figure S3-1. ^1^H NMR spectrum (400MHz, DMSO-*d*_6_) of compound 7c

# Figure S3-2. ^13^C NMR spectrum (101MHz, DMSO-*d*_6_) of compound 7c

# Figure S3-3. HR MS of compound 7c


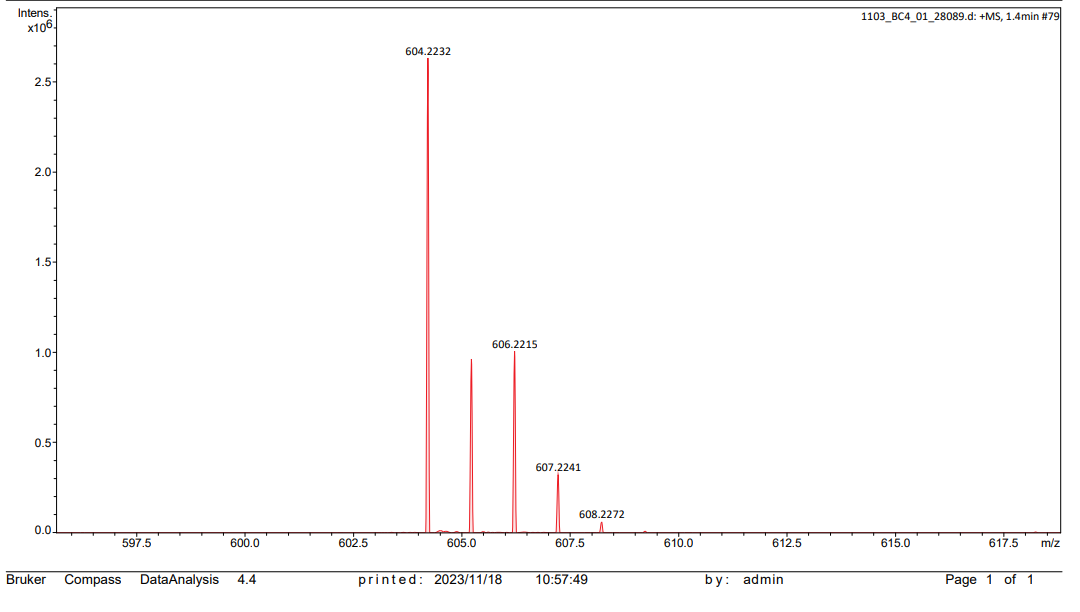


# Figure S4-1. ^1^H NMR spectrum (400MHz, DMSO-*d*_6_) of compound 7d

# Figure S4-2. ^13^C NMR spectrum (101MHz, DMSO-*d*_6_) of compound 7d

# Figure S4-3. HR MS of compound 7d


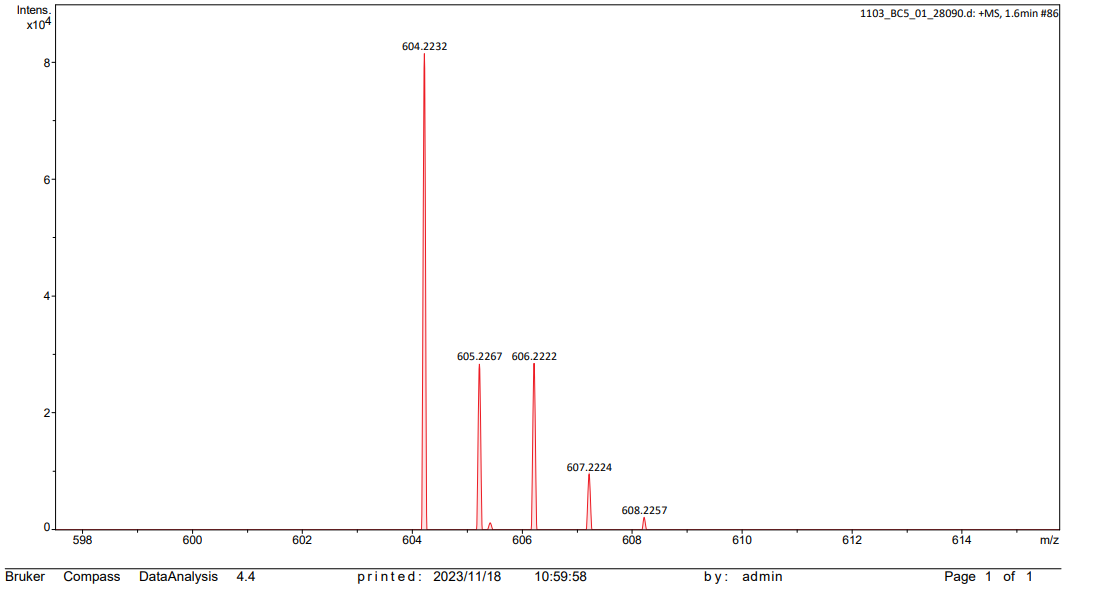


# Figure S5-1. ^1^H NMR spectrum (400MHz, DMSO-*d*_6_) of compound 7e

# Figure S5-2. ^13^C NMR spectrum (101MHz, DMSO-*d*_6_) of compound 7e

# Figure S5-3. HR MS of compound 7e


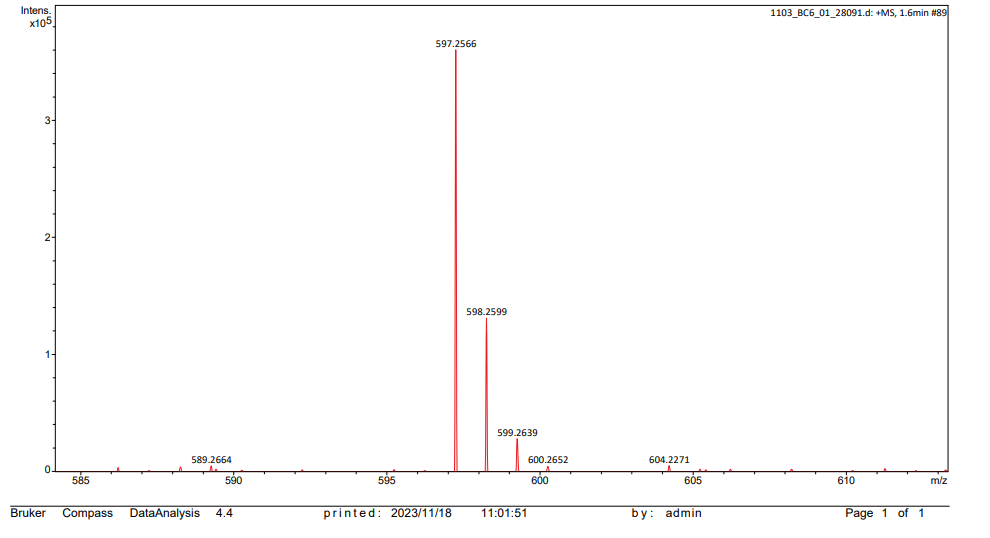


# Figure S6-1. ^1^H NMR spectrum (400MHz, DMSO-*d*_6_) of compound 7f

# Figure S6-2. ^13^C NMR spectrum (101MHz, DMSO-*d*_6_) of compound 7f

# Figure S6-3. HR MS of compound 7f


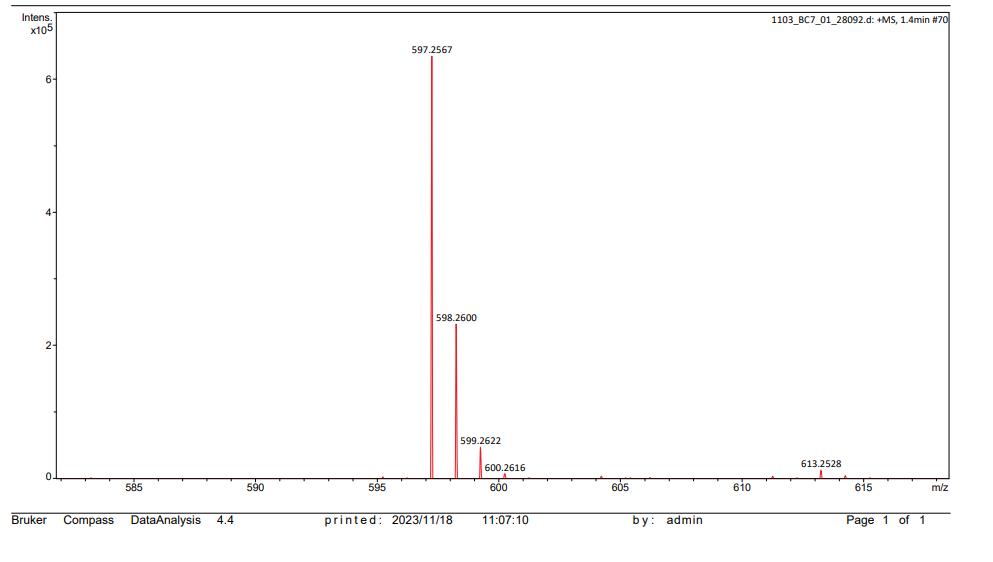


# Figure S7-1. ^1^H NMR spectrum (400MHz, DMSO-*d*_6_) of compound 7g

# Figure S7-2. ^13^C NMR spectrum (101MHz, DMSO-*d*_6_) of compound 7g

# Figure S7-3. HR MS of compound 7g


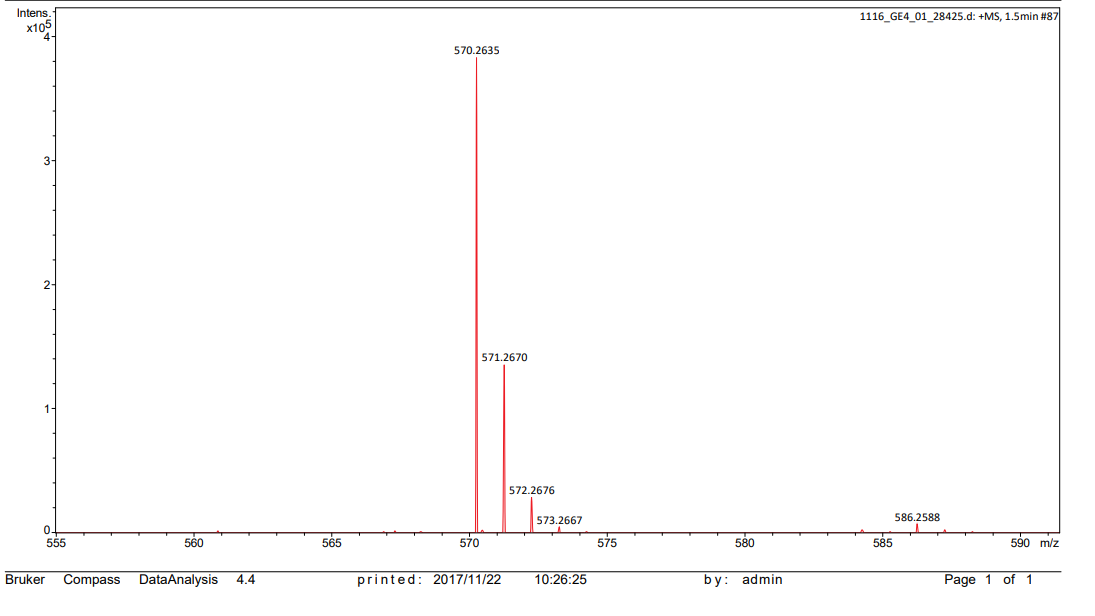


# Figure S8-1. ^1^H NMR spectrum (400MHz, DMSO-*d*_6_) of compound 7h

# Figure S8-2. ^13^C NMR spectrum (101MHz, DMSO-*d*_6_) of compound 7h

# Figure S8-3. HR MS of compound 7h


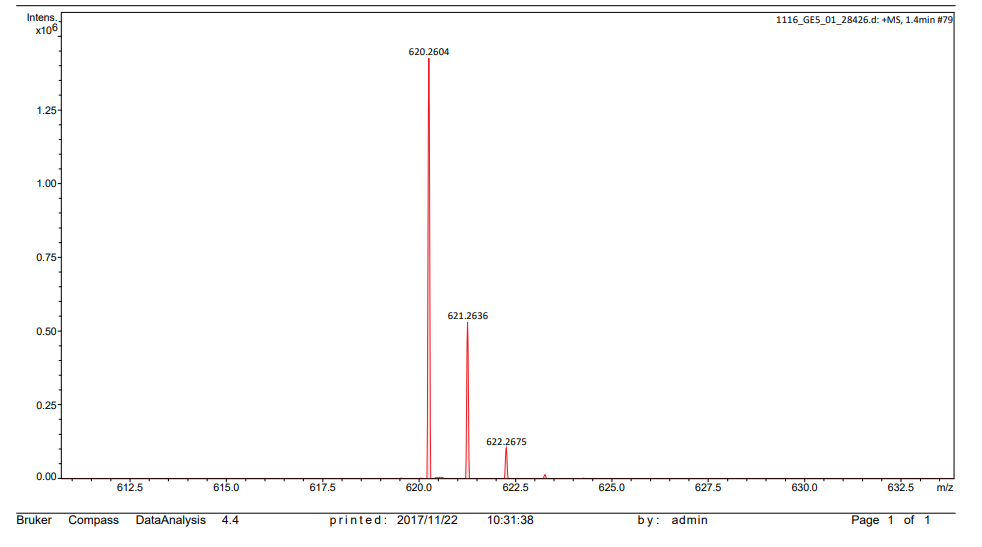


# Figure S9-1. ^1^H NMR spectrum (400MHz, DMSO-*d*_6_) of compound 7i

# Figure S9-2. ^13^C NMR spectrum (101MHz, DMSO-*d*_6_) of compound 7i

# Figure S9-3. HR MS of compound 7i


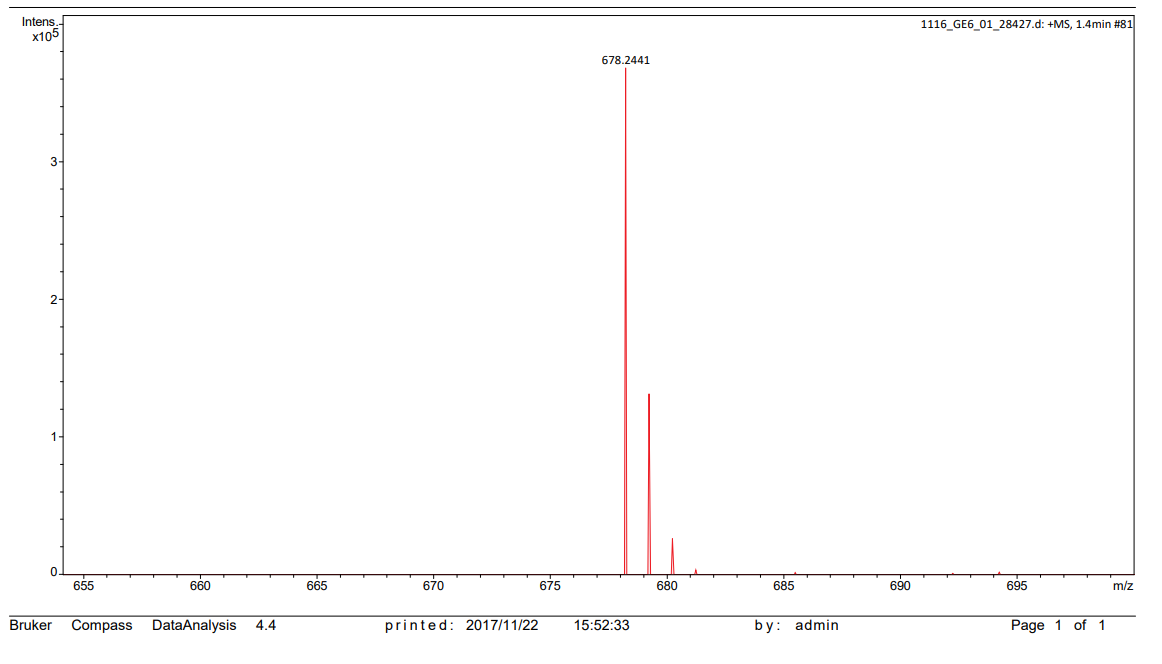


# Figure S10-1. ^1^H NMR spectrum (400MHz, DMSO-*d*_6_) of compound 7j

# Figure S10-2. ^13^C NMR spectrum (101MHz, DMSO-*d*_6_) of compound 7j

# Figure S10-3. HR MS of compound 7j


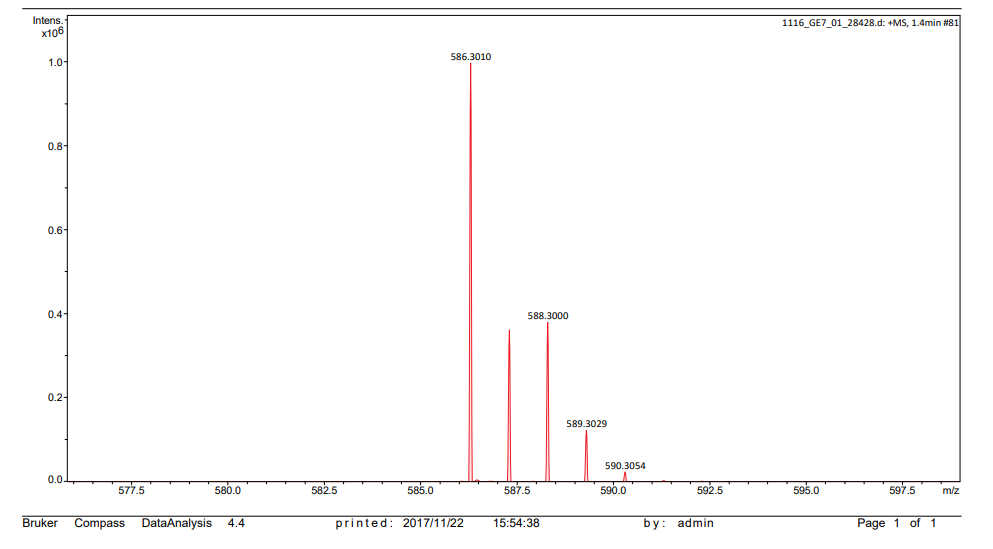


# Figure S11-1. ^1^H NMR spectrum (400MHz, DMSO-*d*_6_) of compound 7k

# Figure S11-2. ^13^C NMR spectrum (101MHz, DMSO-*d*_6_) of compound 7k

# Figure S11-3. HR MS of compound 7k


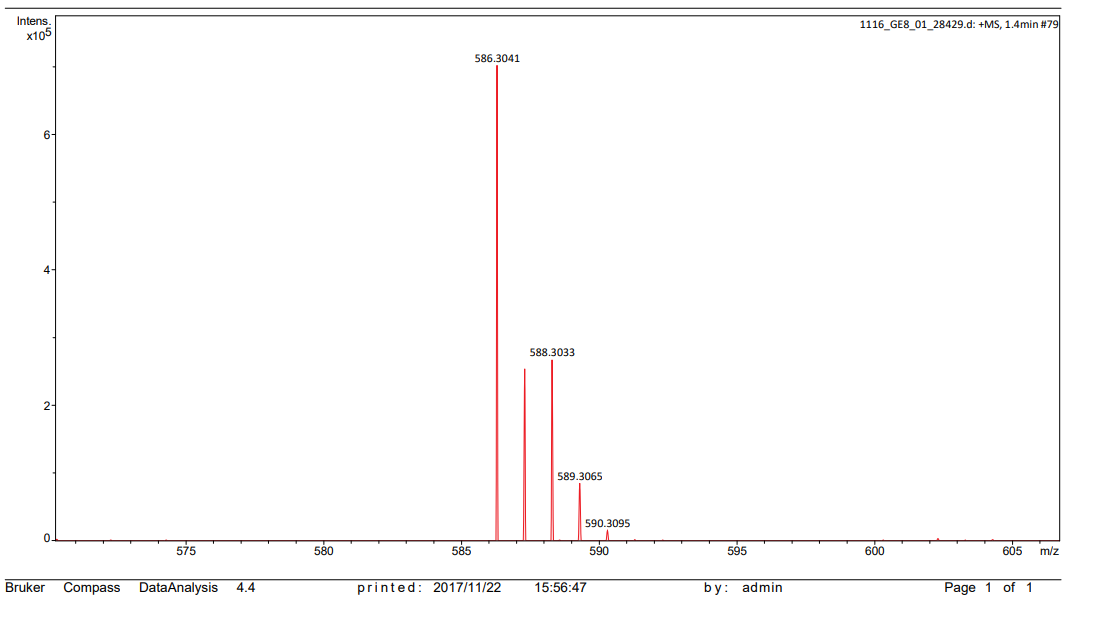


# Figure S12-1. ^1^H NMR spectrum (400MHz, DMSO-*d*_6_) of compound 7l

# Figure S12-2. ^13^C NMR spectrum (101MHz, DMSO-*d*_6_) of compound 7l

# Figure S12-3. HR MS of compound 7l


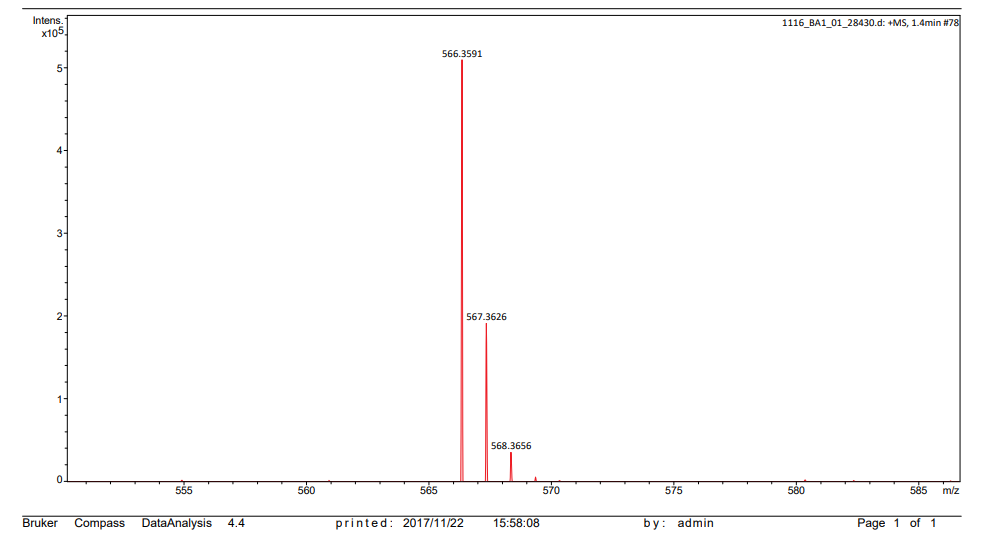


# Figure S13-1. ^1^H NMR spectrum (400MHz, DMSO-*d*_6_) of compound 7m

# Figure S13-2. ^13^C NMR spectrum (101MHz, DMSO-*d*_6_) of compound 7m

# Figure S13-3. HR MS of compound 7m


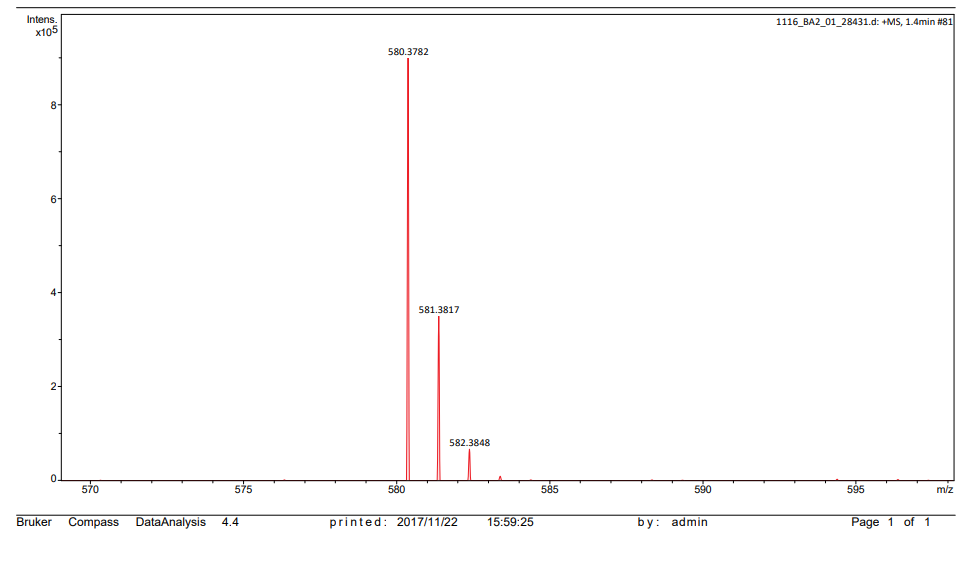


# Figure S14-1. ^1^H NMR spectrum (400MHz, DMSO-*d*_6_) of compound 7n

# Figure S14-2. ^13^C NMR spectrum (101MHz, DMSO-*d*_6_) of compound 7n

# Figure S14-3. HR MS of compound 7n


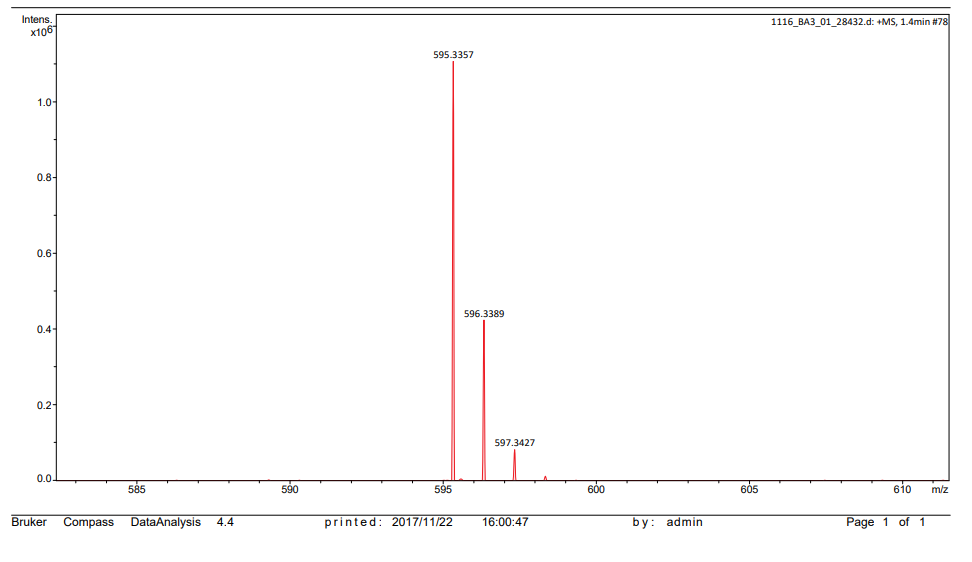


# Figure S15-1. ^1^H NMR spectrum (400MHz, DMSO-*d*_6_ of compound 7o

# Figure S15-2. ^13^C NMR spectrum (101MHz, DMSO-*d*_6_) of compound 7o

# Figure S15-3. HR MS of compound 7o


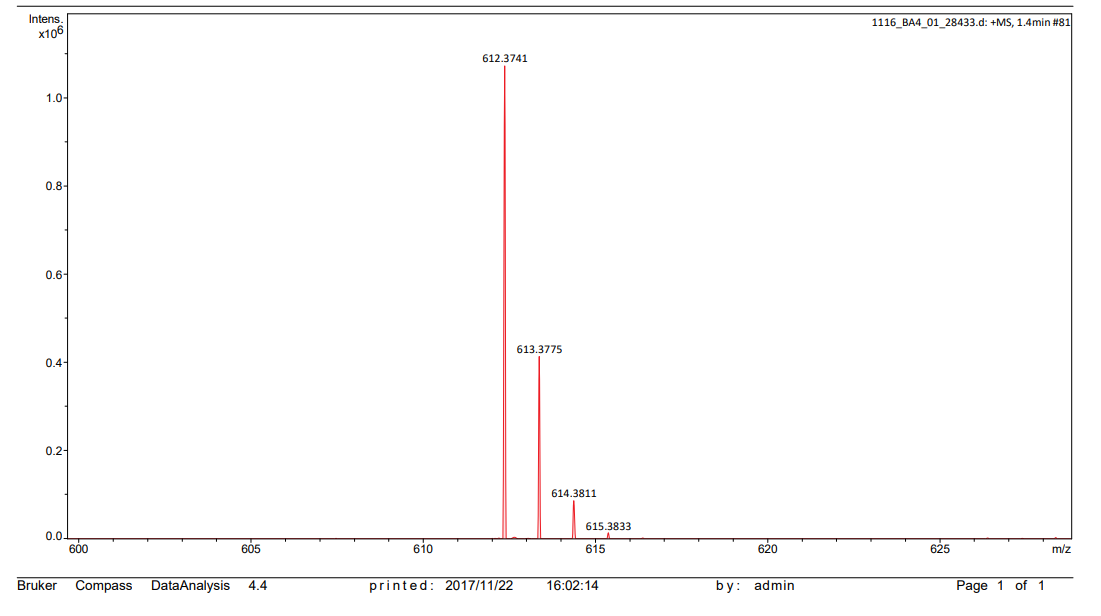


# Figure S16-1. ^1^H NMR spectrum (400MHz, DMSO-*d*_6_) of compound 7p

# Figure S16-2. ^13^C NMR spectrum (101MHz, DMSO-*d*_6_) of compound 7p

# Figure S16-3. HR MS of compound 7p


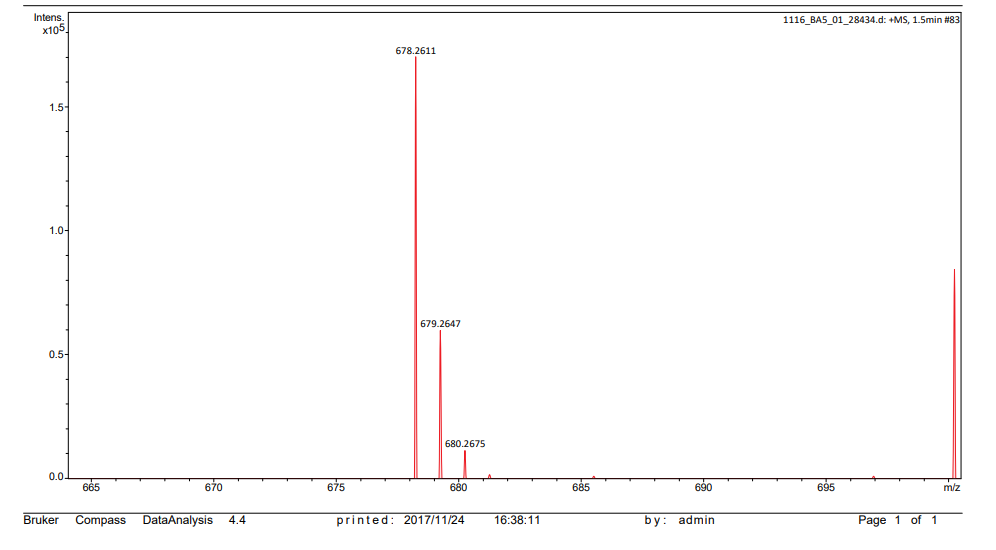


# Figure S17-1. ^1^H NMR spectrum (400MHz, DMSO-*d*_6_) of compound 7q

# Figure S17-2. ^13^C NMR spectrum (101MHz, DMSO-*d*_6_) of compound 7q

# Figure S17-3. HR MS of compound 7q


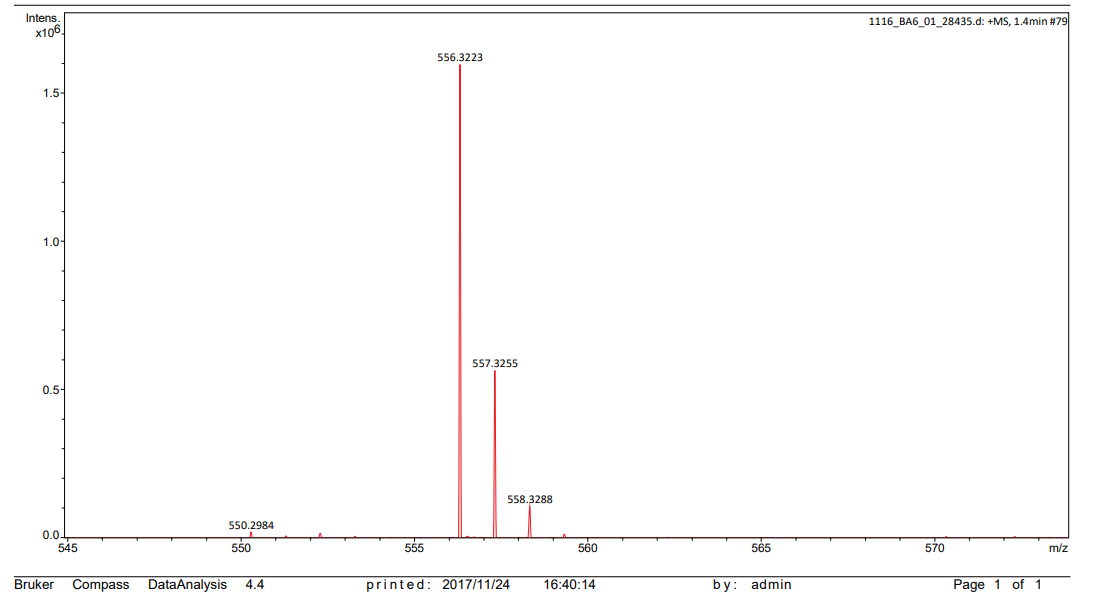


# Figure S18-1. ^1^H NMR spectrum (400MHz, DMSO-*d*_6_) of compound 7r

# Figure S18-2. ^13^C NMR spectrum (101MHz, DMSO-*d*_6_) of compound 7r

# Figure S18-3. HR MS of compound 7r


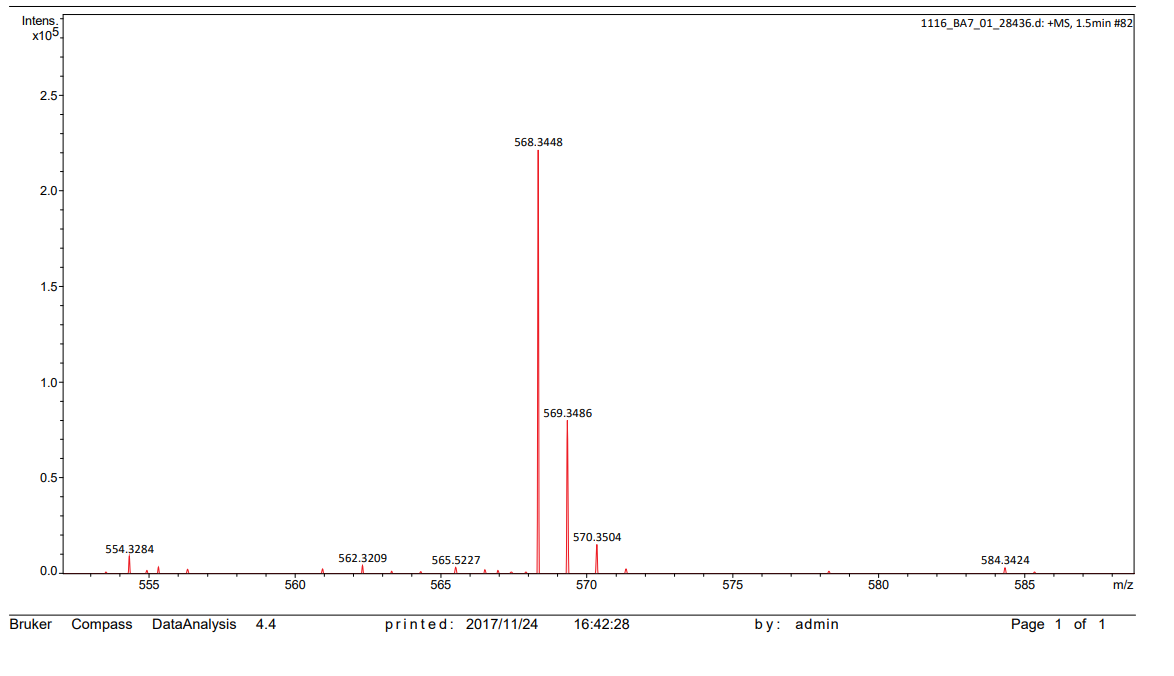


# Figure S19-1. ^1^H NMR spectrum (400MHz, DMSO-*d*_6_) of compound 7s

# Figure S19-2. ^13^C NMR spectrum (101MHz, DMSO-*d*_6_) of compound 7s

# Figure S19-3. HR MS of compound 7s


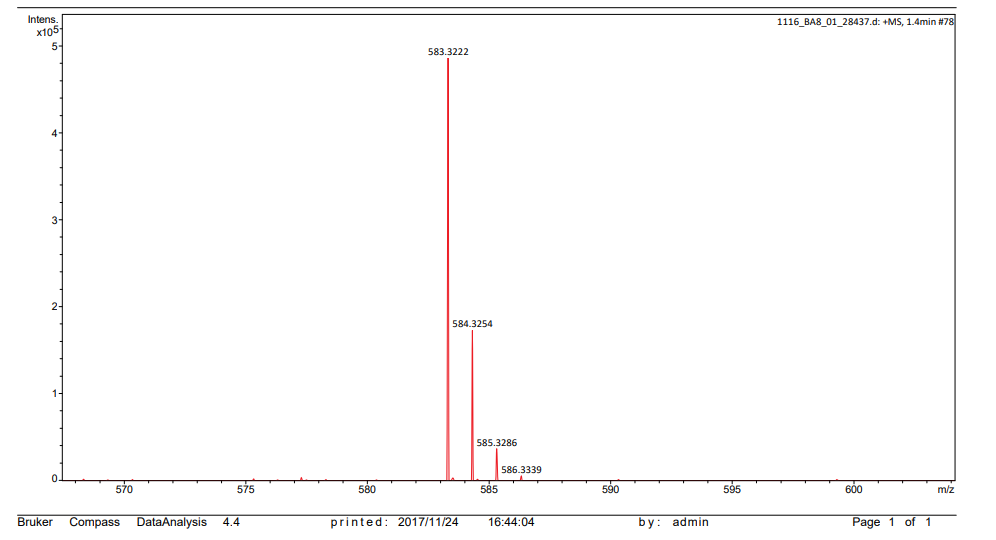


# Figure S20-1. ^1^H NMR spectrum (400MHz, DMSO-*d*_6_) of compound 7t

# Figure S20-2. ^13^C NMR spectrum (101MHz, DMSO-*d*_6_) of compound 7t

# Figure S20-3. HR MS of compound 7t


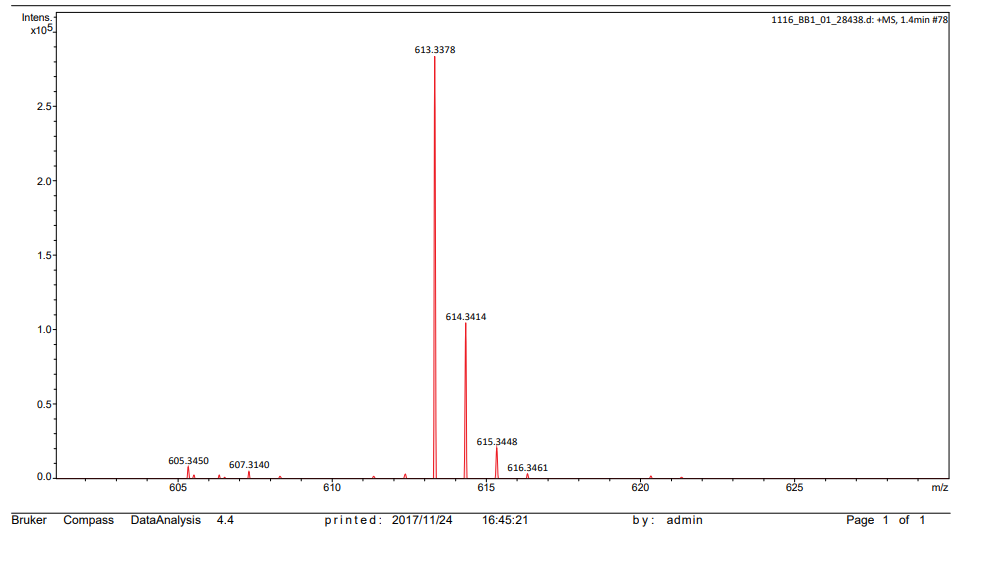

Supplement: Supplementary file 1 — Supplementary Information. [file 41598_2024_60000_MOESM1_ESM.docx]
